# Integrated study of antiretroviral drug adsorption onto calcined layered double hydroxide clay: experimental and computational analysis

**DOI:** 10.1007/s11356-024-33406-7

**Published:** 2024-04-22

**Authors:** Lehlogonolo Shane Tabana, Gbolahan Joseph Adekoya, Shepherd Masimba Tichapondwa

**Affiliations:** 1 Department of Chemical Engineering, Sustainable Environmental and Water Utilisation Processes Division, University of Pretoria, Pretoria, South Africa; 2 Institute of NanoEnginieering Research (INER) & Department of Chemical, Metallurgical and Materials Engineering, Faculty of Engineering and the Built Environment, Tshwane University of Technology, Pretoria, South Africa

**Keywords:** Efavirenz, Nevirapine, Human immunodeficiency virus, Hydrotalcite, Co-precipitation, Emerging pollutants, Memory effect, Molecular modelling

## Abstract

This study focused on the efficacy of a calcined layered double hydroxide (CLDH) clay in adsorbing two antiretroviral drugs (ARVDs), namely efavirenz (EFV) and nevirapine (NVP), from wastewater. The clay was synthesized using the co-precipitation method, followed by subsequent calcination in a muffle furnace at 500 °C for 4 h. The neat and calcined clay samples were subjected to various characterization techniques to elucidate their physical and chemical properties. Response surface modelling (RSM) was used to evaluate the interactions between the solution’s initial pH, adsorbent loading, reaction temperature, and initial pollutant concentration. Additionally, the adsorption kinetics, thermodynamics, and reusability of the adsorbent were evaluated. The results demonstrated that NVP exhibited a faster adsorption rate than EFV, with both reaching equilibrium within 20–24 h. The pseudo-second order (PSO) model provided a good fit for the kinetics data. Thermodynamics analysis revealed that the adsorption process was spontaneous and exothermic, predominantly governed by physisorption interactions. The adsorption isotherms followed the Freundlich model, and the maximum adsorption capacities for EFV and NVP were established to be 2.73 mg/g and 2.93 mg/g, respectively. Evaluation of the adsorption mechanism through computational analysis demonstrated that both NVP and EFV formed stable complexes with CLDH, with NVP exhibiting a higher affinity. The associated adsorption energies were established to be −731.78 kcal/mol for NVP and −512.6 kcal/mol for EFV. Visualized non-covalent interaction (NCI) graphs indicated that hydrogen bonding played a significant role in ARVDs-CLDH interactions, further emphasizing physisorption as the dominant adsorption mechanism.

**Supplementary Information:**

The online version contains supplementary material available at 10.1007/s11356-024-33406-7.

## Introduction

The escalation in the presence of emerging pollutants (EPs), such as pharmaceuticals and personal care products in aquatic environments, is becoming more evident. These substances have the potential to induce detrimental effects on both ecosystems and human health (Caldas et al. 2017; Parolini et al. 2013). Although a comprehensive understanding of the risk assessment and adverse effects associated with these compounds is still lacking, their presence in water bodies raises significant concerns among the general public and regulatory bodies (Schoeman et al. 2015*;* Wooding et al. 2017). Notably, these compounds and their metabolites have been shown to exhibit persistent behavior in the environment due to their limited biodegradability (Mascolo et al. 2010; Prasse et al. 2010). Antiretroviral drugs (ARVDs) are primarily utilized in the treatment of human immunodeficiency virus (HIV) infections, constituting an integral part of antiretroviral therapy (ART). This therapeutic approach aims to suppress viral replication rather than eradicating the virus entirely, thus extending the life expectancy of infected individuals (Ncube et al. 2018). A considerable proportion of orally administered drugs is excreted from the human body after undergoing partial metabolism, ultimately finding their way into water bodies (Tambosi et al. 2010). Furthermore, improper disposal of expired or unused drugs constitutes another pathway through which pharmaceuticals can enter aquatic environments (Insani et al. 2020; Paut et al. 2016).

Efavirenz (EFV) and nevirapine (NVP) are two of the most commonly prescribed ARVDs. These drugs have been detected in the influents and effluents of numerous wastewater treatment plants (WWTPs), as well as in surface and groundwater samples (Abafe et al. 2018; Madikizela et al. 2017; Zitha et al. 2022). The presence of EFV and NVP in WWTPs’ effluents raises concerns regarding the effectiveness of current treatment methods in removing emerging pollutants (EPs). Zitha et al. (2022) stated that the conventional treatment methods of WWTPs implemented in African countries are mainly concentrated on eliminating suspended solids, nutrients, and microorganisms, which explains the presence of pharmaceuticals in effluents. Despite the importance of EFV and NVP in boosting the immune systems of HIV/AIDS patients through ART, their negative impacts may disrupt treatment adherence and lead to drug resistance. The investigation conducted by Ncube et al. (2018) has tied EFV and NVP to hepatotoxicity and neurotoxicity, highlighting their harmful physiological impacts. Studies conducted by Akenga et al. (2021) have revealed that these ARVDs can be absorbed by plants, especially lettuce, suggesting potential unintentional exposure of animals and humans to ARVDs through contaminated crops. Furthermore, studies performed by Fernández et al. (2022) and Kowlaser et al. (2022) have demonstrated the adverse consequences of EFV and NVP on aquatic organisms such as *Rhinella arenarum* tadpoles and *Oreochromis*, respectively. The bioactivity of EPs at minimal concentrations, coupled with the risk of unintended exposure to non-target species from inadequate pharmaceutical disposal and prospective contamination of potable water sources, mandates prompt intervention to mitigate these issues. Adsorption-based processes are favored for removal of ARVDs from wastewater due to their simplicity, minimal energy demands, cost-efficiency, low sludge production, and the possibility of regenerating and reusing adsorbents. (Capra et al. 2018; Yin et al. 2016). In recent years, there has been a growing interest in the development of low-cost and environmentally friendly adsorbents as alternatives to activated carbon. Layered double hydroxide (LDH) clays, characterized by higher surface areas, flexible compositions, and unique structures, have emerged as promising candidates for water remediation in this context. LDH clays can exhibit multidimensional adsorption mechanisms, including anion exchange, surface adsorption, memory effect, and adsolubilization (Lei et al. 2014; Ruan et al. 2013; Tabana et al. 2020; Yang et al. 2016).

Studies by Jie et al. (2022), Kazeem et al. (2019), and Li et al. (2017) have shown that the performance of ternary LDH clays surpasses that of binary LDH clays when employed in water remediation applications. This is due to their improved physicochemical properties, such as enhanced thermal stability, the presence of basic sites, and increased surface area resulting from the synergistic effect between the two metals. Based on these considerations, it was hypothesized that a calcined zinc-containing hydrotalcite (Mg-Al-CO_3_ LDH) would demonstrate effectiveness as an adsorbent for removing EFV and NVP from a simulated wastewater. Therefore, the aim of this study was to investigate the efficacy of calcined Mg-Zn-Al-CO_3_ LDH as an adsorbent for removing EFV and NVP from wastewater. The specific objectives included the synthesis of the adsorbent, followed by a comprehensive characterization utilizing various techniques. Furthermore, adsorption experiments were conducted to investigate the effects of adsorbent loading, operational temperature, initial pH of the solution, and initial concentration of the pollutants. Additionally, the reaction kinetics and adsorption isotherms were established. The adsorbent’s reusability capabilities were evaluated while computational modelling was used to simulate the adsorption mechanisms.

## Experimental

### Materials

The precursor chloride salts, aluminum chloride hexahydrate (AlCl_3_·6H_2_O), magnesium chloride hexahydrate (MgCl_2_·6H_2_O), and zinc chloride (ZnCl_2_), along with solid sodium carbonate (Na_2_CO_3_), were sourced from Glassworld, South Africa. Sodium hydroxide (NaOH) was obtained in solid form from Merck (Pty) Ltd. The ARVDs, EFV and NVP, were procured as solid powders from Adcock Ingram. Methanol (99%) and acetonitrile (99.9%) were used as solvents in the experiments and were provided as solutions by Sigma-Aldrich. Hydrochloric acid (HCl) (32%) was also supplied as a solution by Sigma-Aldrich, while acetic acid (99%) was obtained in liquid form from Glassworld. All materials were used as received without further processing, except for NaOH and HCl, which were diluted prior to use for pH adjustment. Deionized water, dispensed by an Elga Purelab Flex 3 water purifier, was utilized for all the experiments.

### Synthesis and calcination of layered double hydroxide clay

The co-precipitation method was employed to synthesize LDH clay at a constant pH of 10 (± 0.25). The metal salts (Zn, Al, and Mg) were dissolved in deionized water to form a solution with cations having molar ratios of 5%, 20%, and 75%, respectively. Sodium carbonate was used as an anion carrier (CO_3_^2−^), while NaOH was used for pH adjustment. The synthesis mixture was allowed to age for 24 h, after which the precipitates were recovered, washed with deionized water, and dried in an oven at 50 °C for 12 h. The dried precipitates were then pulverized and divided into portions for subsequent analysis, calcination, and adsorption tests. The calcination process was carried out in an aluminum electric muffle furnace at 500 °C. At this temperature, it was anticipated that the LDH clay would undergo a transition, resulting in forming mixed metal oxides (Tabana et al. 2020). These oxides are well-known for their ability to adsorb contaminants from wastewater through multiple mechanisms, such as surface adsorption and adsolubilization. The furnace was preheated to 500 °C, and a clay sample was placed in a porcelain crucible before charging it into the furnace. The temperature was maintained at 500 °C for a duration of 4 h. After completion of the calcination process, the porcelain crucible was removed from the furnace, and the residues were taken for further analysis and adsorption studies.

### Adsorbent characterization

Several techniques were employed to characterize the adsorbent and determine its mineralogy, morphology, thermal phase transitions, functional groups, and Brunauer-Emmett-Teller (BET) surface area. X-ray powder diffraction (XRD) analysis was conducted using a PANalytical X’Pert Pro powder diffractometer in *θ*-*θ* configuration, equipped with an X’Celerator detector and Co-Kα radiation with Fe-filtering. The XRD spectra were collected in the angular range of 5 to 90° 2*θ* with a step size of 0.008° 2*θ* and a scan step time of 13 s. The mineral phases were identified using X’Pert Highscore Plus software, which indexed the spectra against the ICSD database. A Zeiss Ultra Plus field emission scanning electron microscope (FEG-SEM) was used for imaging the morphology. Samples were prepared by distributing them on carbon tape affixed to a microscopy stub, followed by carbon sputter coating under argon gas. The BET surface area was determined using a Micrometrics Tristar 3000 BET analyzer. Before analysis, the samples were degassed for 24 h at 150 °C under a 10^−5^ Torr vacuum. To monitor the thermal phase transitions of the clay, approximately 10 mg of the sample was weighed into alumina crucibles. The crucible containing the sample was then analyzed using the Q5000 Thermogravimetric Analyzer (TGA). The TG analysis involved subjecting the sample to a temperature scan from 25 to 950 °C, with a heating rate of 10 °C per minute. The analysis was carried out under a nitrogen flow rate of 50 ml/min, creating an inert atmosphere during the experiment. Main functional groups and anions in the clay were identified using a PerkinElmer 100 Spectrophotometer. The instrument was equipped with a MIRacle attenuated total reflection (ATR) attachment, which had a zinc-selenide (ZnSe) crystal plate. A powdered sample weighing ca. 20 mg was placed onto the crystal plate and pressed by lowering the pressure arm until the force gauge was ca. 80 before data could be collected. The spectra were recorded between 550 and 4000 cm^−1^ at a resolution of 2 cm^−1^ with data collected over 32 scans.

### Design of experiments

The experimental design and statistical data analysis were performed using Design Expert software (version 13.0, Stat-Ease Minneapolis, USA). Response surface methodology (RSM) with an optimal (custom) design was employed to investigate the four independent variables’ effects on the system. These variables included adsorbent loading (A), operational temperature (B), initial pH of the solution (C), and initial concentration of the pollutant (D). Separate experiments were conducted for EFV and NVP. The optimal design approach was chosen because it allows for any input (numeric, discrete, or categorical) and accommodates any constraints while minimizing the number of experimental runs required for the specified polynomial model. In this study, 21 experimental runs were conducted to optimize the levels of the design factors. The ranges for the variables were as follows: adsorbent loading (A) ranged from 5 to 20 g/L, operational temperature (B) ranged from 25 to 60 °C, initial pH of the solution (C) ranged from 5 to 12, and initial pollutant concentration (D) ranged from 5 to 20 mg/L. The experimental data were fitted to a quadratic model for the statistical analysis, as represented by Eq. (1). The response variable of interest was the adsorption efficiency (%), determined according to Eq. (2). The residence time for all 21 runs was kept constant at 24 h.

1 $$Y={P}_0+\sum_{i=1}{P}_i{x}_i+{\sum}_{i=1}^k{\sum}_{j=1}^k{P}_{ij}{x}_i{x}_j{\sum}_{i=1}^k{P}_{ii}{x}_{ii}+\varepsilon$$

2 $$Y=\left(1-\frac{C_t}{C_0}\right)\times 100$$

where *Y* represents the response variable; *P*_0_ is the intercept; *P*_*i*_, *P*_*ij*_, and *P*_*ii*_ are the coefficients of the linear effect and double interactions; *x*_*i*_ and *x*_*j*_ are the independent variables; *ɛ* is the error; and *C*_*t*_ and *C*_0_ are the pollutant concentration at time *t* and the initial concentration in milligrams per liter, respectively.

### Adsorption studies

Batch experiments were carried out to investigate the adsorption of ARVDs using CLDH as an adsorbent. A volume of 100 mL of simulated wastewater containing the desired concentration of either EFV or NVP was brought into contact with a known mass of CLDH to achieve the desired adsorbent loading. Prior to contact, the solution’s initial pH was adjusted using 0.1 M HCl (acidic) or NaOH (basic). The resulting suspensions were placed on the magnetic stirrers with temperature control, and continuous stirring was maintained for 24 h at the targeted operational temperature to ensure maximum adsorption. After the 24 h runs, the suspensions were filtered through a 0.45-μm Millipore filter. The solution samples obtained from the above-mentioned experiments were analyzed using a Waters Alliance 2695 high-performance liquid chromatography (HPLC). The instrument was equipped with a UV-Vis detector and an auto-sampling unit. Separation occurred in a Waters PAH C18 column, while data was collected using Empower software. Elution was done through a mobile phase consisting of acetonitrile, methanol, and a pH 4.5 buffer solution through a gradient flow. Statistical analysis was performed on the data obtained from the 21 runs to determine the optimum operational conditions. Additional tests were conducted to investigate the adsorption kinetics, isotherms, thermodynamics studies, and reusability of the adsorbent.

### Computational method

**Fig 1:**
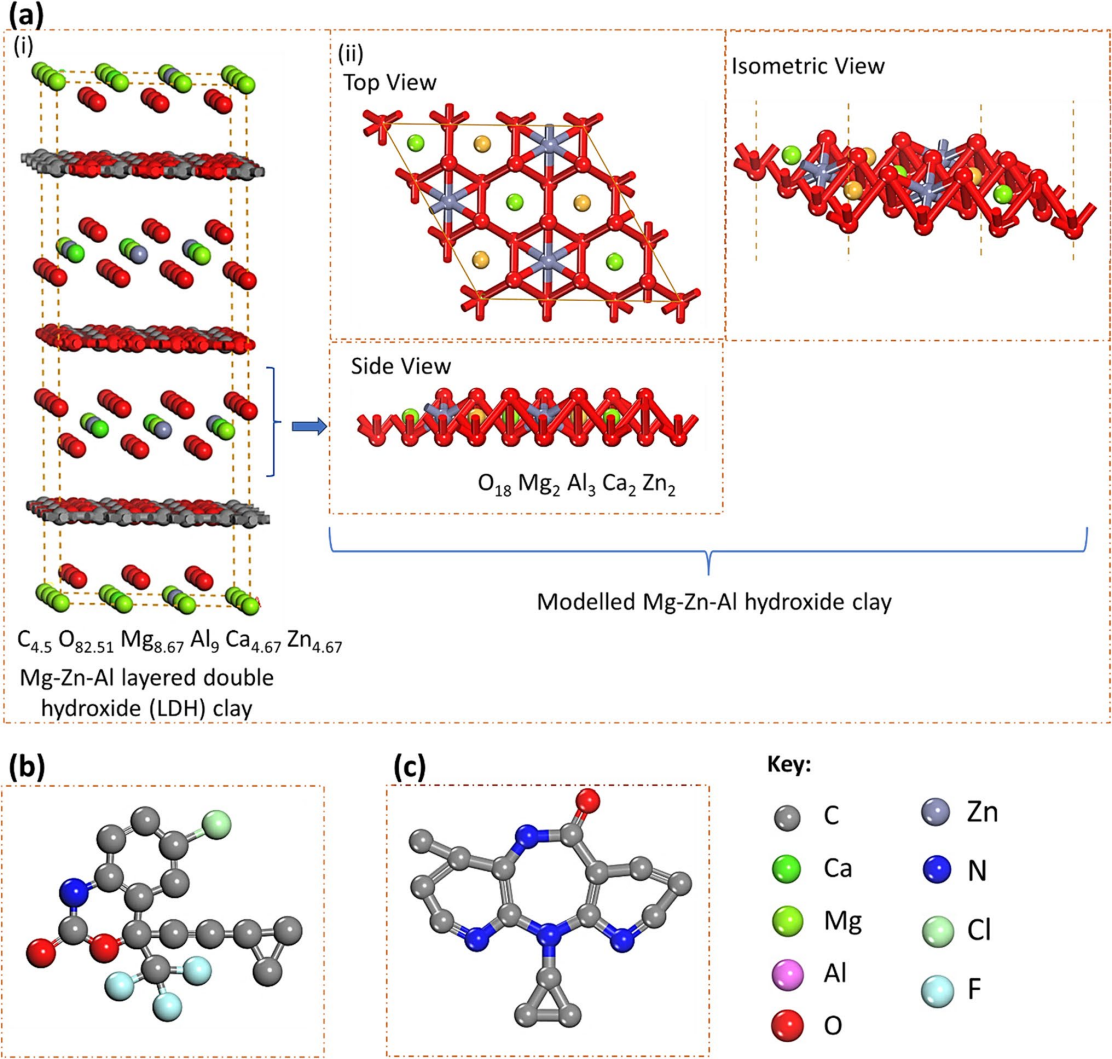
3D structure of the **a** (i) 3 × 3 CLDH (C4.5 O82.51 Mg8.67 Al9 Ca4.67 Zn4.67) showing the (ii) top, side, and isometric views of the modelled section of the CLDH, **b** EFV and **c** NVP

Materials Studio 2020 software was used to analyze the adsorption and non-covalent interactions (NCI) of EFV and NVP on CLDH clay. Figure 1 shows the modeled structures of the adsorbates and adsorbent generated using the builder module within the software. The lattice parameters of the 3 × 3 supercell of CLDH were *a* = 9.13 Å and *b* = 9.13 Å with a vacuum of *c = *20 Å for the adsorption of the ARVDs. The Adsorption Locator module was used for geometry optimization and adsorption annealing simulation (Adekoya et al. 2022). This module employs COMPASSIII forcefield for geometry relaxation and the Monte Carlo method to explore various configurational spaces and predict the most stable binding locations of the drugs on the CLDH.

The procedure involved gradually lowering the system’s temperature as the drug molecules adsorbed onto the clay. This allowed for the determination of local energy minima, indicating the most thermodynamically favorable configurations. The probability of accepting a selected configuration was determined using Eq. (3), while the adsorption energy was estimated by the Adsorption Locator using Eq. (4) (Adekoya et al. 2023).

3 $${P}_{\textrm{mn}}=\min \left(1,\frac{\uprho_n}{\uprho_m}\right)$$

4 $$\Delta E={E}_{\textrm{sorbate}/\textrm{substrate}}-\left({E}_{\textrm{sorbate}}+{E}_{\textrm{substrate}}\right)$$

where *ρ*_*m*_ represents the frequency of sampled m configurations, *ρ*_*n*_ represents the frequency of suggested n configurations, and *P*_*mn*_ denotes the likelihood of a configuration transition from *m* to *n*. Δ*E* represents the binding energy of the adsorbed atom to CLDH (kcal/mol). *E*_sorbate/substrate_ is the energy of the system per cell (kcal/mol)*,* while *E*_sorbate_ and *E*_substrate_ represent the isolated energies for the sorbate and adsorbate in the supercell (kcal/mol), respectively.

NCIs were computed using the Multiwfn software. Scatter plots were generated using gnuplot, and the visual representation of the plots was facilitated by visual molecular dynamics (VMD) software. Various types and strengths of interactions, including steric repulsion and strong attraction, can be identified through the analysis of isosurfaces and the reduced density gradient (RDG). RDG was calculated using Eq. (5) (Adekoya et al. 2022).

5 $$RDGs=\frac{1}{2{\left(3{\pi}^2\right)}^{\frac{1}{3}}}\frac{\left|\overline{\Delta \rho }(r)\right|}{{\overline{\rho (r)}}^{\frac{4}{3}}}$$

Here, *ρ*(*r*) is the electron density, and ∆*ρ*(*r*) is the gradient norm of electron density.

## Results and discussion

### Adsorbent characterization

#### X-ray diffraction (XRD) analysis

**Fig. 2:**
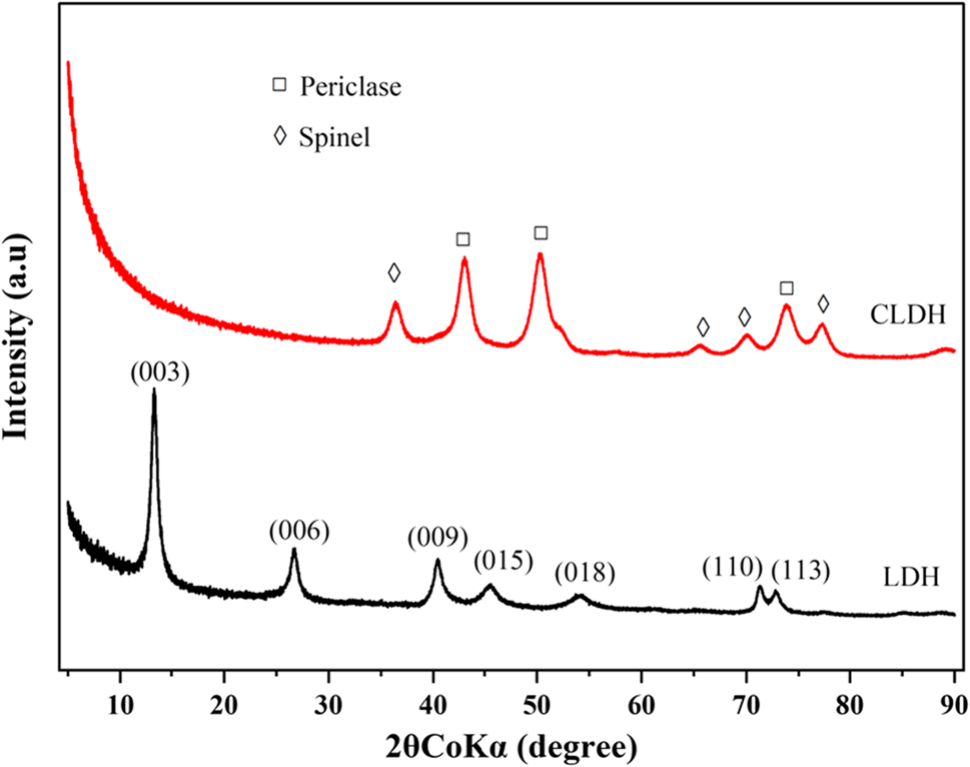
X-ray diffraction spectra of neat and calcined LDH clays

The XRD spectra of the neat LDH and CLDH clays are shown in Fig. 2. The neat LDH clay exhibited characteristic peaks that were clearly identified and assigned to specific crystal planes. The amorphous-dominated CLDH displayed two phases that were identified as periclase (MgO) and spinel (MgAl_2_O_4_). The quantitative analysis of CLDH indicated that it contained 55% periclase and 45% spinel. Dos Santos et al. (2017) and Gao et al. (2013) revealed that of the two phases; the periclase phase played a key role in the adsorption process. A substantial amount of this phase holds promising potential for the adsorption of ARVDs from wastewater.

#### Thermogravimetric analysis

**Fig. 3:**
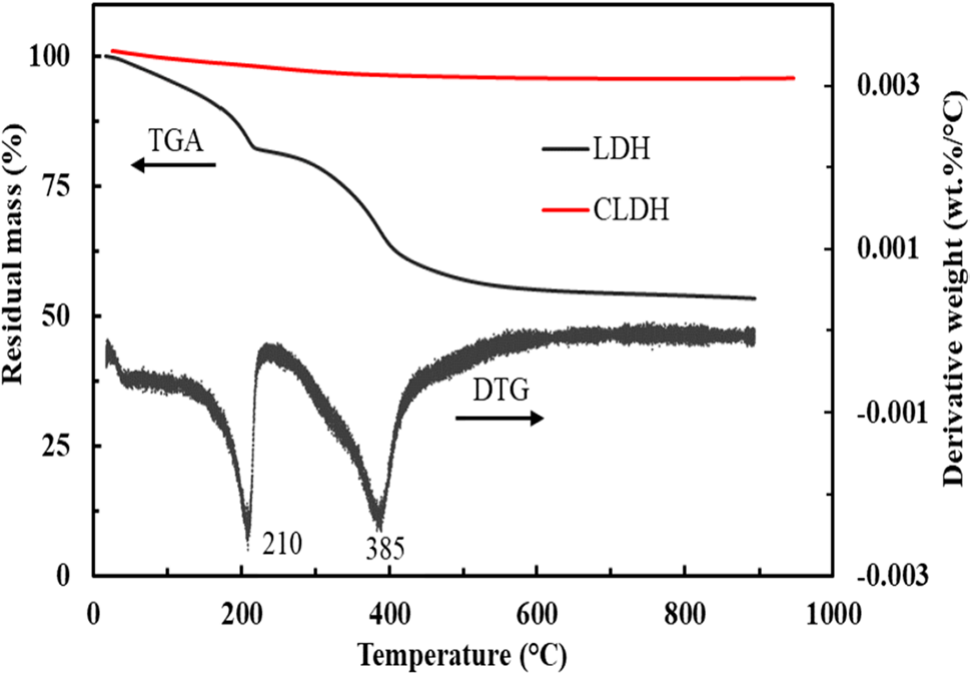
Thermogravimetric analysis of neat and calcined LDH clay

Figure 3 shows the thermal analysis of the neat LDH clay and CLDH. Thermal analysis of the neat LDH displayed two distinct decomposition stages, a characteristic synonymous with LDH clays with an M^2+^/M^3+^ ratio of 2. The first decomposition stage occurred at 210 °C and was accompanied by a mass loss of 18%. This weight loss can be attributed to the loss of physiosorbed water and a partial loss of hydroxyl ions (OH^−^), which are an integral part of the LDH structure. The second decomposition stage took place at 385 °C, resulting in a mass loss of 25%. This stage is associated with the interlayer ions’ complete decomposition in the LDH structure. These decompositions transformed a highly crystalline LDH clay into an amorphous-dominated mixed metal oxide (MMO). The thermal analysis of CLDH indicated a minimal loss of 3% throughout the evaluated temperature range. The negligible loss can be attributed to the removal of surface moisture, as the material was anticipated to completely transform into MMO during the calcination process.

#### Brunauer-Emmett-Teller (BET) surface area

**Fig. 4:**
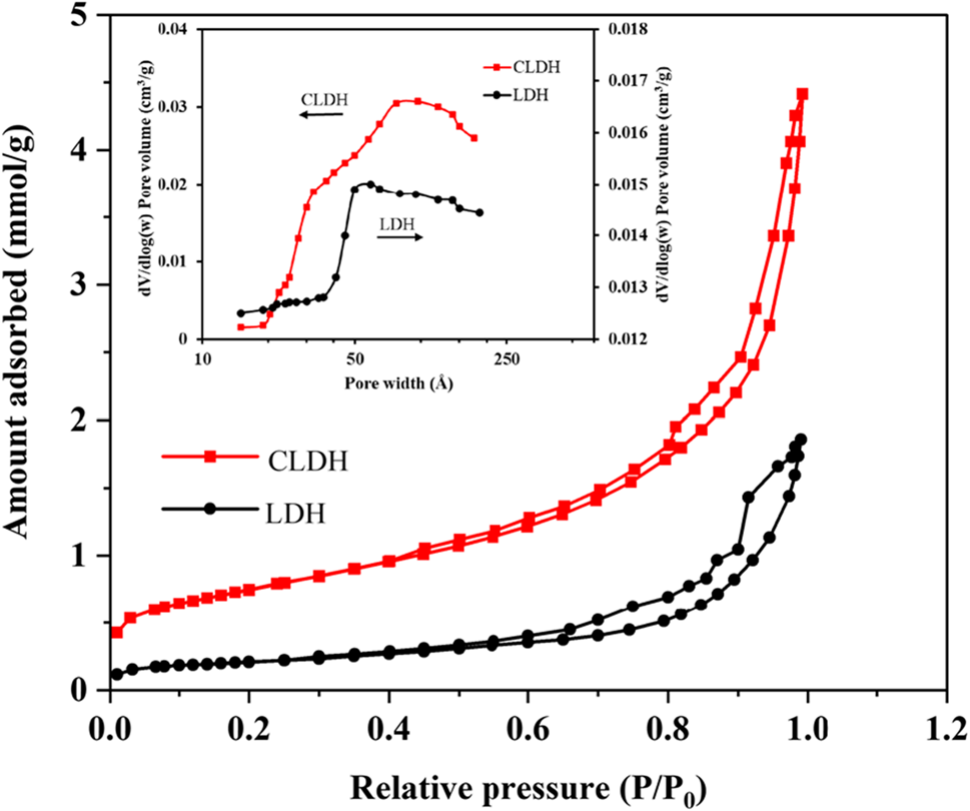
BET surface area analysis for neat LDH and CLDH

Figure 4 illustrates the nitrogen gas adsorption and desorption isotherms for both neat LDH and CLDH. The isotherms demonstrated a distinct sorption behavior of Type IV, characterized by a hysteresis loop of type H3, as per the IUPAC (Li et al. 2016). The estimated parameters for the BET surface area, pore volume, and average pore diameter from the neat LDH were 28.5 m^2^/g for the BET surface area, 80.6 Å for the average pore diameter, and a pore volume of 0.155 cm^3^/g. The pore size distribution curve showed a broad peak between 2 and 100 nm, with a maximum at 5 nm, indicating the presence of mesopores/macropores in the product, possibly related to pores created between hexagonal plates. CLDH had a BET surface area of 90.4 m^2^/g, an average pore diameter of 44.3 Å and a pore volume of 0.344 cm^3^/g. The pore size distribution curve, like that of neat LDH, showed a noticeable peak within the 2 to 100 nm range, peaking at 10 nm, indicating the presence of mesopores/macropores, presumably due to the pores generated between hexagonal plates.

#### Fourier transform infrared (FTIR) analysis

**Fig. 5:**
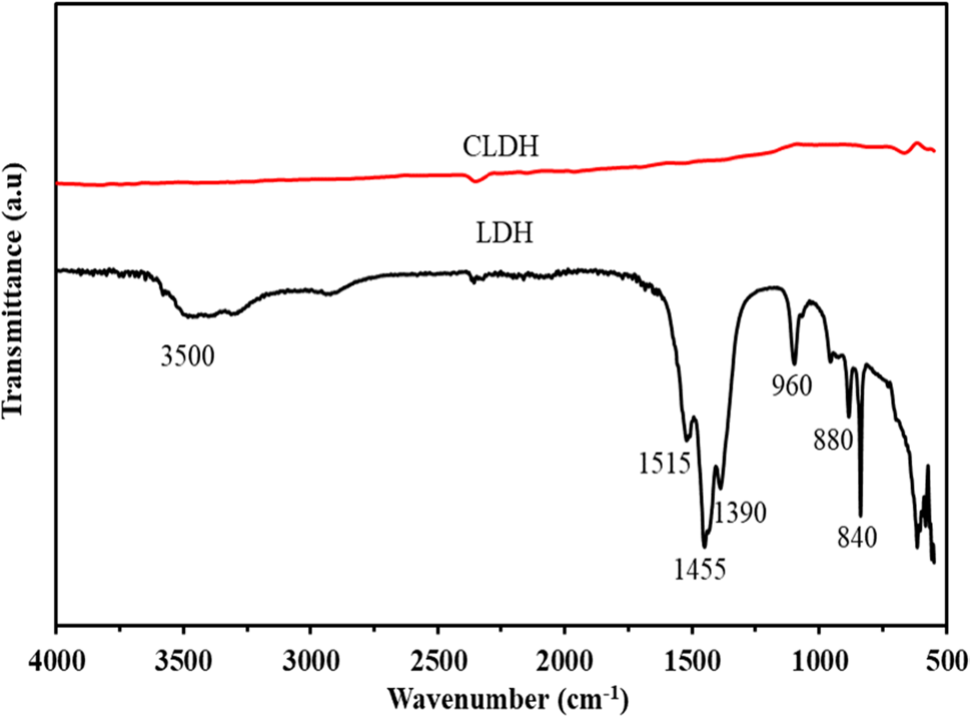
FTIR analysis for neat LDH clay and CLDH

Figure 5 presents the infrared spectra obtained for the neat LDH clay and CLDH. The neat LDH spectra revealed distinct bands associated with specific vibrational modes and molecular interactions within the clay. The band observed at 840 cm^−1^ can be attributed to the ν1 vibrational modes of carbonate ions (CO_3_^2−^), while the peak at 880 can be related to ν2 of the same ions. The band at 960 cm^−1^ corresponded to the vibrational modes of metal-O-metal linkages, signifying the presence of bonds between the metal cations within the layered structure of the clay. Furthermore, the ν1 vibrational mode of CO_3_^2−^ is represented by a weaker band at 1100 cm^−1^, indicating the asymmetric stretching of CO_3_^2−^ ions.

Additionally, a band at 1390 cm^−1^ indicated the presence of CO_3_^2−^ ions in the interlayer region of the LDH structure. The bands observed within the range of 1515 and 1455 cm^−1^ were attributed to the vibration of OH^−^, confirming their presence in the LDH clay. The peak at 3500 cm^−1^ was ascribed to stretching the O–H bond associated with water molecules in the interlayers and metal hydroxide layers (Li et al. 2016; Naseem et al. 2019). Calcination of the clay resulted in the disappearance of the main spectra, which corresponded with XRD results indicating structural changes and the transformation of the clay to MMO.

#### Scanning electron microscopy (SEM) analysis

**Fig. 6:**
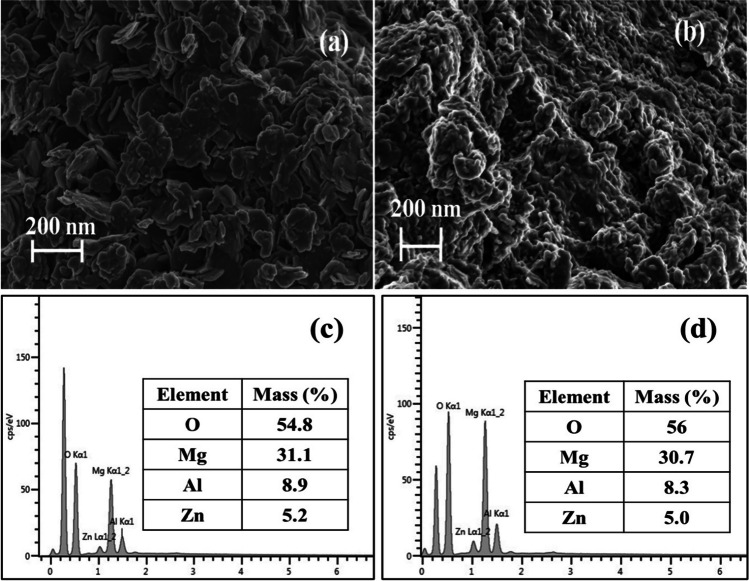
SEM and SEM-EDS analysis for neat LDH clay (**a**, **c**) and CLDH (**b**, **d**)

Figure 6 a and b present the morphological analysis of the neat and calcined LDH (CLDH) clays using SEM. For the neat LDH clay, the SEM images exhibit a hexagonal plate-like morphology of the particles. This particular structure aligns with previous reports in the literature concerning LDH clays based on magnesium and aluminum (Naseem et al. 2019). The observed hexagonal platelets reflected the well-ordered arrangement of layers within the LDH structure. Conversely, upon subjecting the LDH clay to calcination at 500 °C, noticeable changes in morphology occurred. The SEM images of the CLDH illustrated the absence of ordered hexagonal platelets observed in the neat LDH. Instead, the particles demonstrated an irregular and less defined morphology, indicating the structural transformation during the calcination process. This transformation led to the formation of irregular particles composed of MMO. The platelet assemblies displayed in Fig. 6a had an estimated size of 200 nm, as demonstrated by the scanning electron micrographs. Scherrer’s equation was used to estimate particle sizes, with two orientations considered: one parallel to the LDH layers and another perpendicular to the LDH layers, as explained by Gevers et al. (2019). The estimated crystallite sizes in perpendicular and parallel dimensions were 12 and 20 nm, respectively. This substantiates the presence of nano-sized particles in the clay, further validating the material’s nanoscale properties. The elemental compositions of both the neat and CLDH materials were analyzed utilizing energy dispersive X-ray spectroscopy (EDS), with the findings depicted in Fig. 6 c and d, respectively. The conducted elemental analysis affirmed the existence of Mg, Zn, and Al within both materials.

**Fig. 7:**
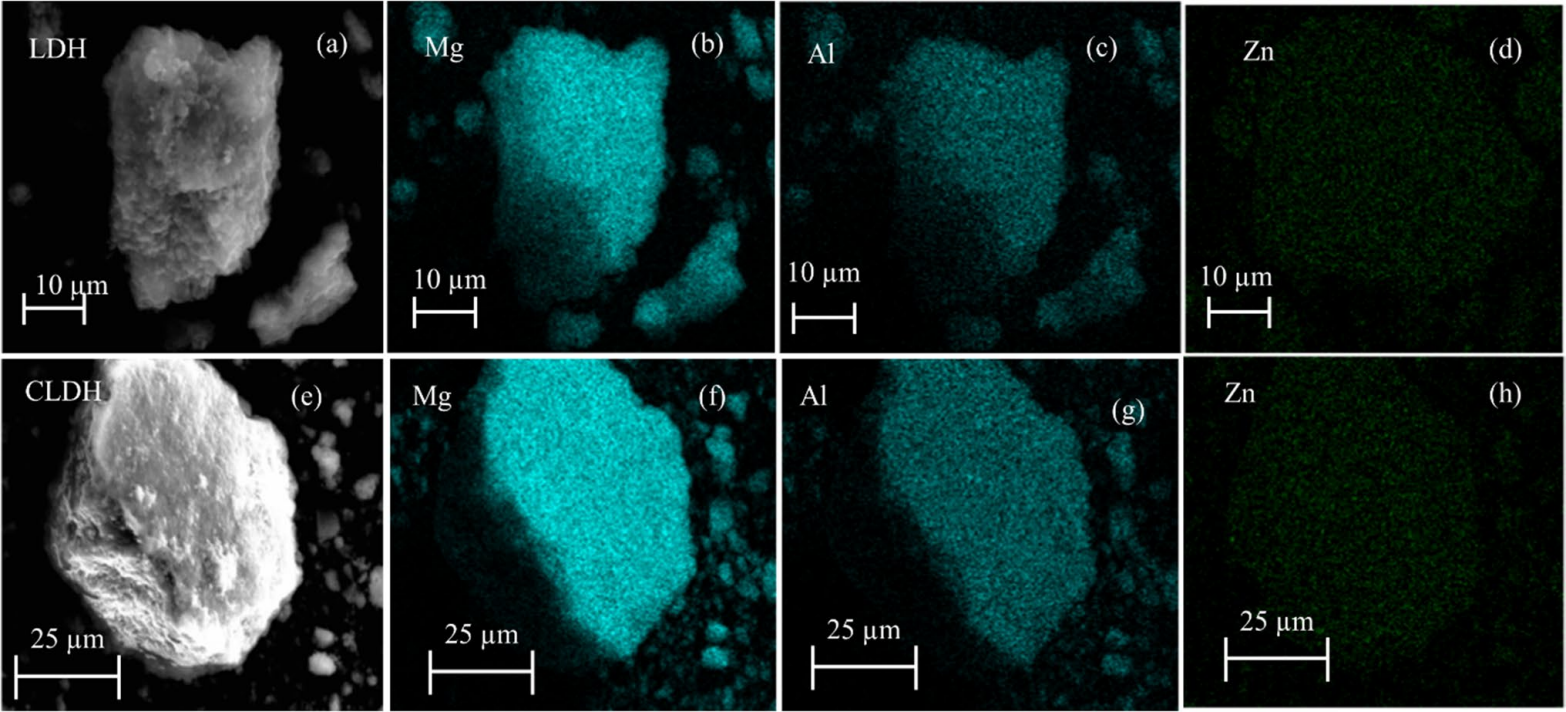
HAADF-STEM image of neat LDH (**a**), EDS mapping (neat LDH), Mg (**b**), Al (**c**), Zn (**d**), HAADF-STEM image of CLDH (**e**), EDS mapping (CLDH), Mg (**f**), Al (**g**), Zn (**h**).

Figure 7 shows the high-angle annular dark-field scanning transmission electron microscopy (HAADF-STEM) image as well as the energy-dispersive X-ray spectroscopy (EDS) elemental mapping images for both the neat LDH clay (Fig. 7a–d) and the CLDH (Fig. 7e–h). The HAADF-STEM images (Fig. 7a and e) provide visual representations of the neat LDH clay and CLDH, respectively, revealing the spatial distribution and arrangement of various elements within the materials. The elemental maps (Fig. 7b–d and f–h) depict the presence and distribution of Mg, Al, and Zn.

### Adsorption results

#### Statistical analysis of the adsorption process using response surface methodology

**Fig. 8:**
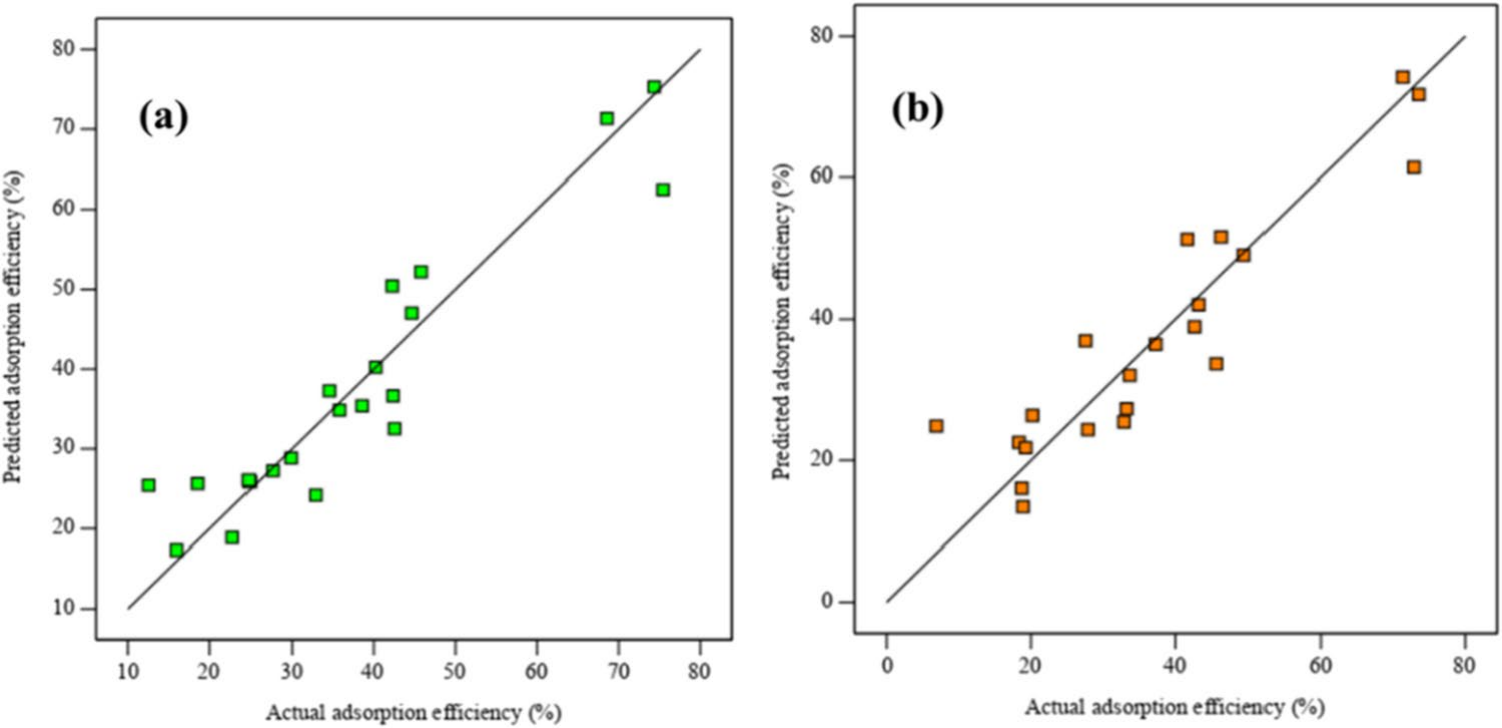
Predicted adsorption efficiency vs actual adsorption efficiency EFV (**a**) and NVP (**b**)

RSM was employed to assess the comparative importance of the experimental parameters, utilizing a combination of mathematical and statistical analysis techniques. A quadratic equation was adjusted to the experimental responses obtained from the designed experiments, and an analysis of variance (ANOVA) was performed. Table 1 presents the different combinations of independent variables and their corresponding responses (adsorption efficiency) for both EFV and NVP from the 21 experimental runs. The significance of the model equation was evaluated using the *F*-test for ANOVA. The ANOVA statistics for EFV and NVP adsorption efficiencies are displayed in Tables 2 and 3, respectively. The *p*-values for the adsorption efficiency in both tables were found to be lower than 0.05, indicating that the model was statistically significant. The minimal values of the standard deviations (4.62 and 5.61 for EFV and NVP, respectively) between the predicted and experimental results indicated that the model equations (Eqs. (6) and (7)) accurately represented the realistic relationship between the significant variables and the responses. Higher values of *R*^2^ (0.877 and 0.898 for EFV and NVP, respectively) and predicted *R*^2^ (0.811 and 0.854 for EFV and NVP, respectively) further confirmed a strong dependence and correlation between the predicted and observed values. These observations are also visually depicted in Fig. 8a and b, which compare the predicted values from the respective models with the experimental ones. Given the linear trend demonstrated by the data points on the plots, it can be deduced that the residuals follow a normal distribution, hence negating the need for data transformation (Alimohammady et al. 2017). Therefore, it can be concluded that the prediction of the experimental data acquired from the quadratic model for the adsorption of EFV and NVP by CLDH is reasonably satisfactory.

6 $$Y=25.64-3.99A-5.07B+0.13C-15.35D+11.14 AC+4.87 BD+16.45{D}^2$$

7 $$Y=26.36-5.49A-5.54B-17.05D+11.97 AC+4.02 BD+14.74{D}^2$$

**Table 1:** Experimental runs and the corresponding adsorption efficiencies for EFV and NVP

Run	*A*: adsorbent loading (g/L)	*B*: temperature (°C)	*C*: pH	*D*: pollutant concentration (mg/L)	Efavirenz adsorption efficiency (%)	Nevirapine adsorption efficiency (%)
1	20	25	5	20	16.2	18.9
2	10	65	9	5	44.7	49.5
3	5	25	9	10	40.2	43.2
4	12.5	45	7	12.5	18.6	20.2
5	5	65	12	20	22.8	18.7
6	5	45	12	5	42.3	46.3
7	10	25	5	5	68.6	73.6
8	10	25	12	15	32.9	27.9
9	20	25	12	5	74.3	71.4
10	20	65	5	5	42.4	37.3
11	15	55	12	20	29.9	32.9
12	12.5	45	5	10	42.5	45.7
13	5	35	5	20	34.6	27.6
14	20	25	12	20	35.8	33.7
15	20	55	9	20	24.8	19.3
16	5	65	5	10	38.6	42.7
17	20	45	7	5	45.8	41.7
18	12.5	65	5	20	24.9	18.3
19	20	65	12	12.5	27.7	33.3
20	20	45	9	12.5	12.5	6.9
21	20	35	9	5	75.4	72.9

**Table 2:** Analysis of variance for the quadratic model (EFV)

Source	Sum of squares	DF	Mean square	*F*-value	*P*-value
Model	5375.75	7	767.96	13.25	< 0.0001
*A*—adsorbent loading	168.01	1	168.01	2.9	0.1124
*B*—temperature	410.67	1	410.67	7.09	0.0196
*C*—pH	0.1389	1	0.1389	0.0024	0.9617
*D*—pollutant concentration	3298.93	1	3298.93	56.92	< 0.0001
*AC*	646.05	1	646.05	11.15	0.0053
*BD*	203.68	1	203.68	3.51	0.0084
*D*^2^	1011.59	1	1011.59	17.45	0.0011
Residual	753.45	13	57.96		
Lack of fit	17.05	5	3.41	0.7056	0.6587
Pure error	14.5	3	4.83		
Cor total	6129.2	20

**Table 3:** Analysis of variance for the quadratic model (NVP)

Source	Sum of squares	DF	Mean square	*F*-value	*P*-value
Model	5900.99	6	983.5	13.24	< 0.0001
*A*—adsorbent loading	320.53	1	320.53	4.32	0.0567
*B*—temperature	390.51	1	390.51	5.26	0.0379
*D*—pollutant concentration	4105.01	1	4105.01	55.26	< 0.0001
*AC*	746.89	1	746.89	10.06	0.0068
*BD*	144.22	1	144.22	1.94	0.0019
D^2^	813.16	1	813.16	10.95	0.0052
Residual	1039.9	14	74.28		
Lack of fit	305.47	6	50.91	8.13	0.569
Pure error	18.8	3	6.27		
Cor total	6940.89	20

#### Interpretation of 3-dimensional (3D) and contour plots

**Fig. 9:**
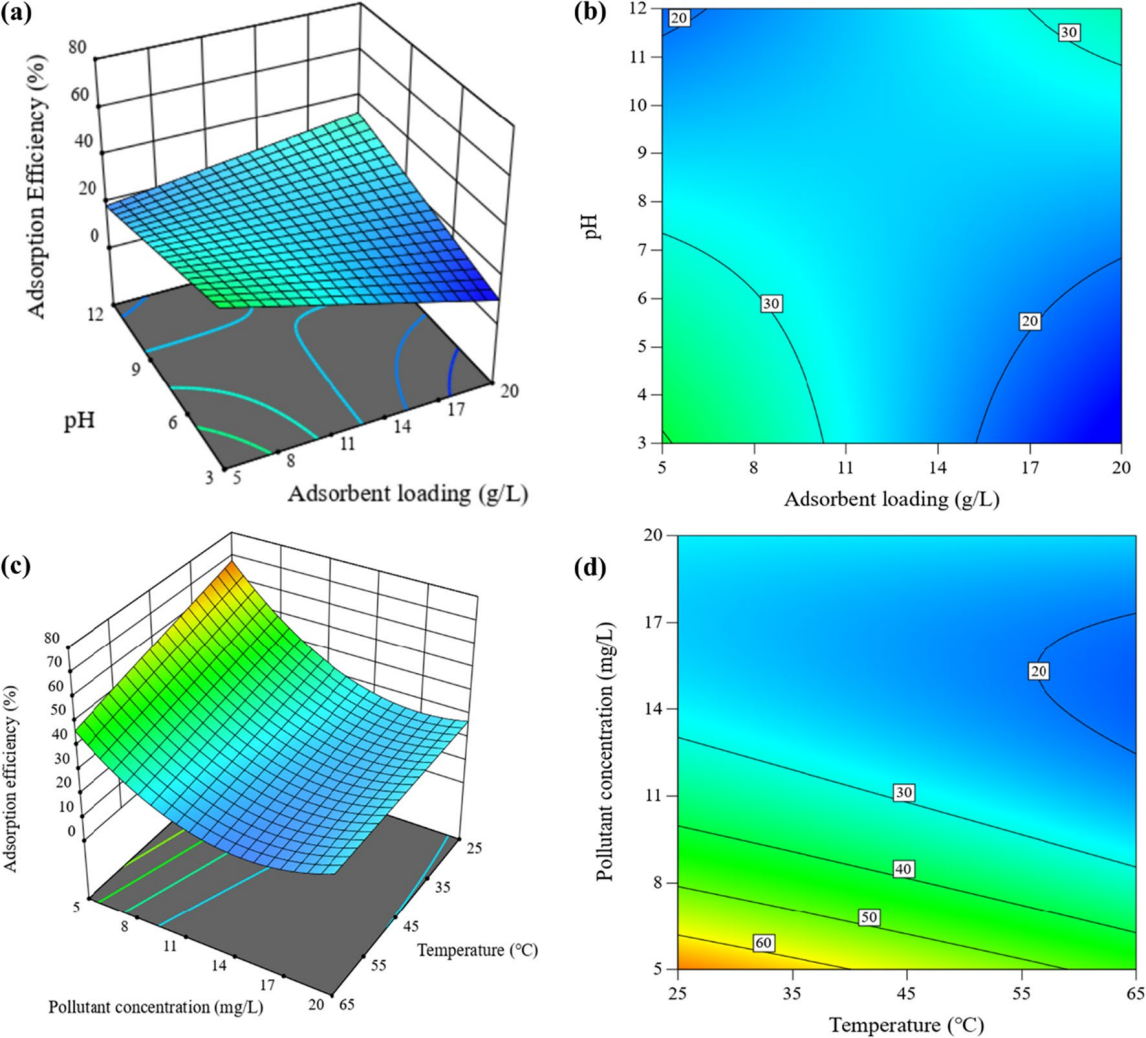
Response surface plots (3D and contour projection) showing variables’ effects on adsorption of EFV. **a**, b Adsorbent loading (5–20 g/L) vs pH (5–20); **c**, **d** temperature (25–65 °C) vs pollutant concentration (5–20 mg/L)

**Fig. 10:**
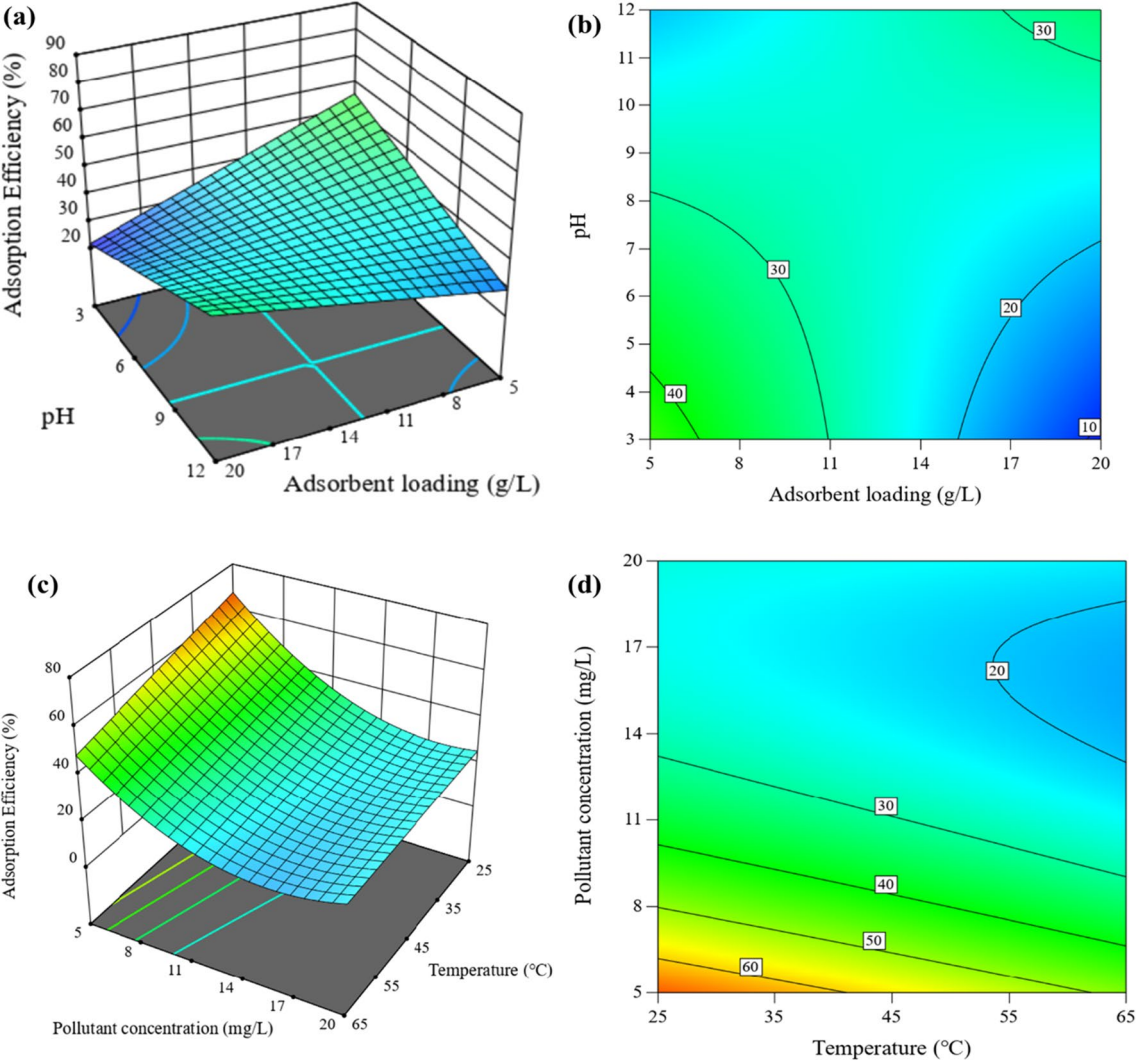
Response surface plots (3D and contour projection) showing variables’ effects on adsorption of NVP. **a**, **b** Adsorbent loading (5–20 g/L) vs pH (5–20); **c**, **d** temperature (25–65 °C) vs pollutant concentration (5–20 mg/L)

Figures 9 and 10 depict the 3D plots and contour projections for the interactions between pH and adsorbent loading, along with pollutant concentration, and temperature, for both EFV and NVP. The temperature versus pollutant concentration projections were similar for both pollutants, indicating a consistent trend. However, there was a slight variation in the projections for pH versus adsorbent loading, particularly in the contour plot for EFV, where the 40% adsorption efficiency percentile was not clearly defined.

The temperature versus pollutant concentration projections demonstrated that higher adsorption efficiencies can be achieved at lower pollutant concentrations and temperatures. The explanation of this occurrence can be attributed to two primary factors. Firstly, as pollutant concentrations decrease, the number of active sites accessible for interaction increases. This enhanced availability of active sites allows for more effective pollutant adsorption. Secondly, at lower temperatures, molecular species have less mobility, making them more susceptible to being captured by clay particles (Chabi et al. 2020; Oliveira et al. 2008).

The interactions between pH and adsorbent loading revealed that high adsorption efficiencies can be obtained under two conditions: low pH and low adsorbent loading, as well as high pH and high adsorbent loading. The pKa value of NVP is 2.8, indicating that the molecule has a positive charge at pH levels below 2.8 and becomes neutral at pH values over 2.8. Similarly, the point of zero charge for CLDH is 8.9, as shown in Figure S1 in the supplementary data. Consequently, conducting the adsorption process at a pH range of 2.8 to 8.9 was expected to promote NVP removal due to electrostatic interactions with CLDH. EFV has a pKa value of 10.2, suggesting that the molecule is positively charged at pH values lower than 10.2. This means that electrostatic interactions between EFV and CLDH will be influenced by the clay’s pH-neutralizing capacity, which is regulated by adsorbent loading. At pH greater than 12, the reconstruction of CLDH may be accelerated due to the increased concentration of OH^−^ (Clark et al. 2019; Eiby et al. 2016). Both molecules (EFV and NVP) would be neutral under these conditions, whereas the clay would be negatively charged. As a result, weak electrostatic interactions would occur, leading to reduced removal efficiencies.

Moderate adsorbent loadings are favored as they provide an adequate number of active sites while minimizing the risk of reduced dispersion efficiency and agglomeration of the adsorbent, which can block active sites (Tabana et al. 2020). Acidic pH conditions are not recommended for processes involving LDH clays, as they can potentially damage the clay structure (dos Santos et al. 2013). However, this negative impact can be mitigated by operating at optimum adsorbent loadings, as the clay can neutralize the solution due to its natural pH being between 8 to 10 (Amamra et al. 2021; Elhachemi et al. 2022). Based on these results, the optimal conditions were determined to be a pH of 5, an adsorbent loading of 10 g/L, and an ambient temperature of approximately 25 °C.

#### Reaction kinetics and the effect of pollutant concentration

**Fig. 11:**
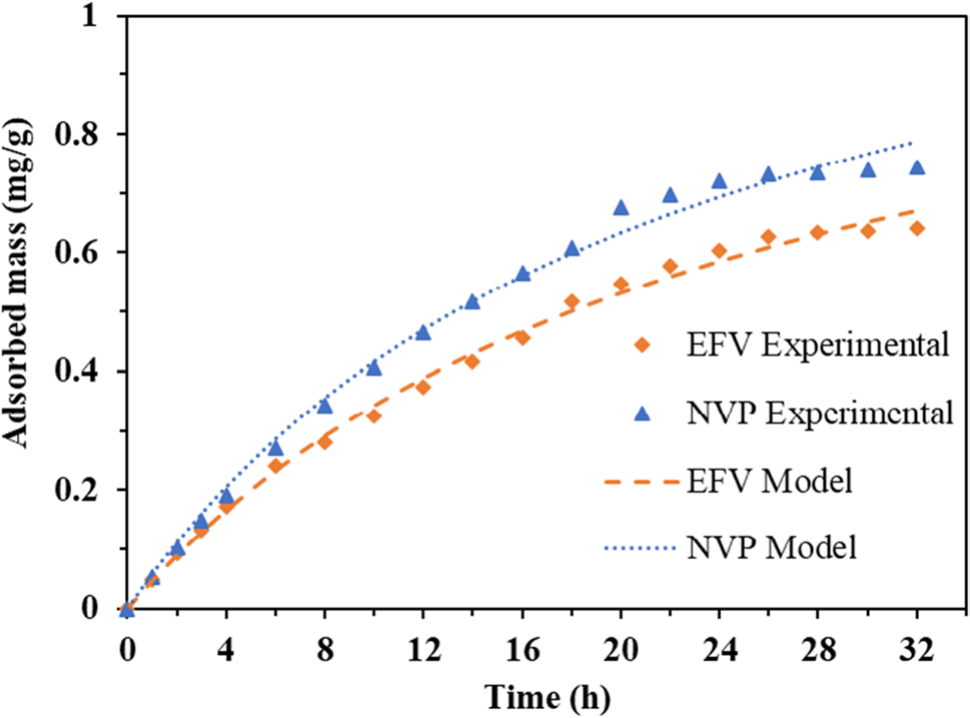
Adsorbed amounts of EFV and NVP and PSO kinetics model for EFV and NVP at an initial concentration of 10 mg/L, pH 5, *T* = 25 °C, and adsorbent loading of 10 g/L

Figure 11 displays the amounts of EFV and NVP adsorbed by a CLDH as determined through Eq. (8). The results indicated that NVP exhibited a faster adsorption rate compared to EFV, reaching equilibrium after 20 h, whereas EFV required 24 h to reach equilibrium. The kinetic data was fitted to the pseudo-first order (PFO) and pseudo-second order (PSO) kinetics models, as represented by Eqs. (9) and (10). The kinetics data was found to be fitting onto the PSO model as depicted in Fig. 11 for both pollutants with an initial concentration of 10 mg/L (the fitted kinetics at 5 mg/L and 20 mg/L for EFV and NVP are shown in Figures S2 and S3 in the supplementary data). Detailed kinetics parameters for EFV and NVP adsorption are displayed in Table 4 (PFO parameters are shown in Table S1 in the supplementary data).

8 $${q}_t=\frac{V}{m}\left({C}_0-{C}_t\right)$$

9 $${q}_t={q}_e\left(1-{e}^{-{k}_1t}\right)$$

10 $${q}_t=\frac{q_e^2{k}_2t}{q_e{k}_2t+1}$$

where *V* is the volume of wastewater solution (L); *m* is the mass of adsorbent (g); *q*_*e*_ is the mass adsorbed at equilibrium (mg/g); *k*_1_ is the PFO rate constant (h^−1^); and *k*_2_ is the PSO rate constant (g/mg·h).

**Table 4:** PSO kinetic models’ parameters (initial concentration of 10 mg/L, pH 5, *T* = 25 °C, and adsorbent loading of 10 g/L)

Concentration	Parameter	Efavirenz	Nevirapine
5 mg/L	*k*_2_ (g/mg·h)	0.073	0.016
	*q*_*e*_ (calculated)(mg/g)	0.647	1.23
	*q*_*e*_ (experimental)(mg/g)	0.362	0.42
	*R*^2^	0.998	0.997
10 mg/L	*k*_2_ (g/mg·h)	0.034	0.028
	*q*_*e*_ (calculated)(mg/g)	1.19	1.32
	*q*_*e*_ (experimental)(mg/g)	0.629	0.748
	*R*^2^	0.996	0.995
20 mg/L	*k*_2_ (g/mg·h)	0.017	0.006
	*q*_*e*_ (calculated)(mg/g)	1.62	2.81
	*q*_*e*_ (experimental)(mg/g)	0.64	0.824
	*R*^2^	0.997	0.997

The rate constants for both EFV and NVP showed a decrease with an increase in initial concentration, indicating a complex reaction governed by numerous factors, including initial concentration, molecular collisions, or reaction mechanisms (Soustelle 2011). The calculated equilibrium capacities were established to be consistently higher than the experimental values. These disparities demonstrated the model’s inadequacies in fully describing the system’s actual adsorption kinetics. The consistent increase in calculated adsorption capacities indicated a divergence from the PSO kinetic model’s assumptions, particularly those relating to monolayer adsorption and uniform adsorption sites (Guo and Wang 2019; Zhang 2019). These variations indicated that the adsorption mechanisms driving the interaction of EFV and NVP with CLDH were more complex than a monolayer adsorption process and involved non-uniform adsorption sites. Based on these findings, it is evident that the adsorption process is a multidimensional mechanism.

**Fig. 12:**
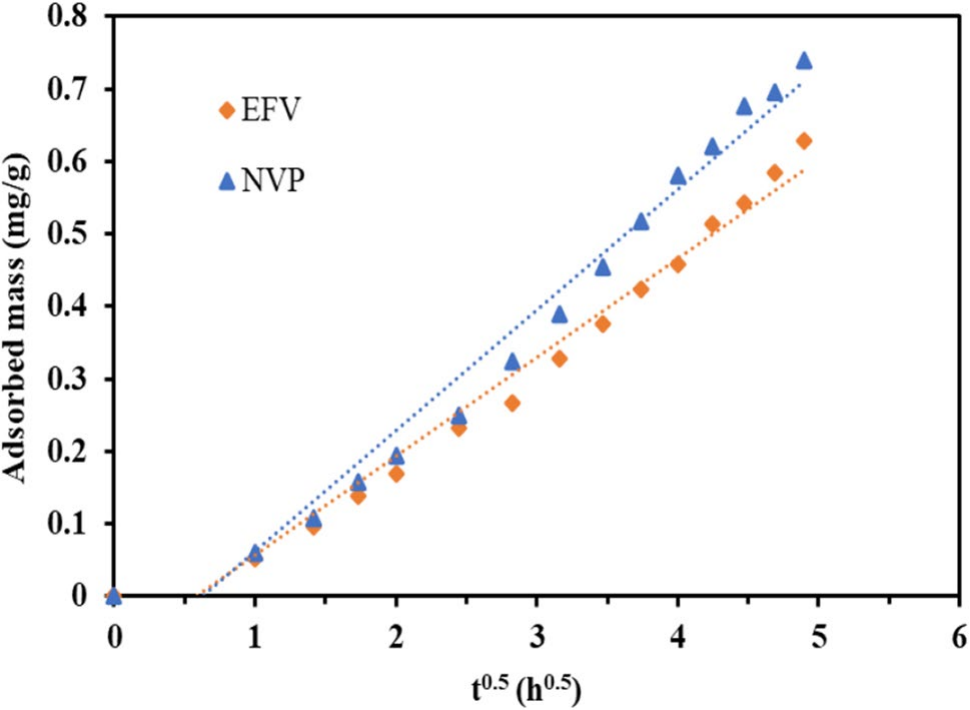
Intra-particle diffusion plots for EFV and NVP at an initial concentration of 10 mg/L, pH 5, T=25 °C, and an adsorbent loading of 10 g/L

The investigation of the intra-particle behavior in the adsorption processes was carried out using Eq. (11), and the corresponding results are presented in Fig. 12. Since the linearized trendlines deviated from passing through the origin, it indicated that intra-particle diffusion alone was not the sole rate-limiting step in the adsorption mechanism (Santi, 2012). Therefore, it can be deduced that both surface adsorption and intra-particle diffusion processes operate concurrently during the interaction between ARVDs and CLDH.

11 $${q}_t={K}_{ip}{t}^{0.5}+C$$

where *K*_*ip*_ is the rate constant of intra-particle diffusion and *C* is the vertical axis intercept.

#### Adsorption isotherms

**Fig. 13:**
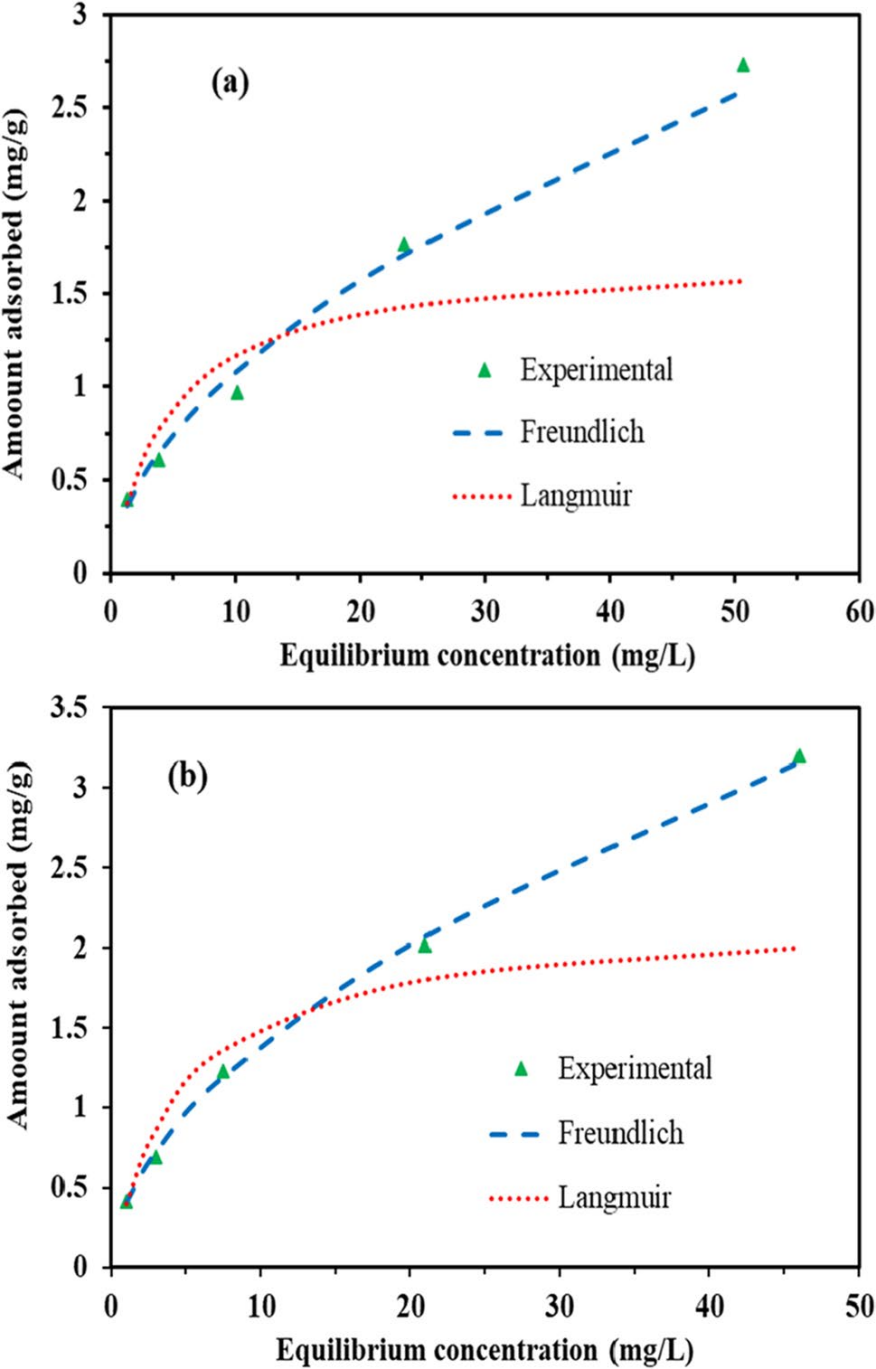
Equilibrium adsorption isotherms **a** EFV and **b** NVP at various concentrations, pH 5, *T* = 25 °C, and an adsorbent loading of 10 g/L

Figure 13 a and b show the adsorption isotherms for both EFV and NVP, respectively, with the adsorption isotherm parameters presented in Table 5. The data was fitted onto the Langmuir and Freundlich isotherms (as shown in Eqs. (12) and (13), respectively), with the latter providing the best fit for both pollutants. Notably, the heterogeneity factor (*n*) exceeds 1 under the tested conditions, indicating favorable adsorption characteristics. The maximum adsorption capacities obtained for EFV and NVP were estimated to be 2.73 mg/g and 2.93 mg/g, respectively. These values are relatively low compared to the results reported by Adeola et al. (2021), who achieved maximum adsorption capacities of 4.41 mg/g and 48.31 mg/g for EFV and NVP, respectively, using graphene wool as the adsorbent.

12 $${q}_e=\frac{q_m{K}_L{C}_e}{1+{K}_L{C}_e}$$

13 $${q}_e={K}_F{C}_e^n$$

where *K*_*F*_ is the Freundlich equilibrium constant (L/g); *K*_*L*_ is the Langmuir constant (L/mg); *C*_*e*_ is the pollutant concentration at equilibrium (mg/L); *n* is the heterogeneity constant (dimensionless); and *q*_*m*_ is the maximum adsorption capacity (mg/g).

**Table 5:** Adsorption equilibrium isotherms parameters (pH 5, *T* = 25 °C, and an adsorbent loading of 10 g/L)

Isotherm	Parameters	Efavirenz	Nevirapine
Langmuir	*K*_*L*_ (L/mg)	0.21	0.48
	*q*_*m*_ (mg/g)	1.71	1.52
	*R*^2^	0.79	0.80
Freundlich	*K*_*F*_ (L/g)	0.31	0.44
	*n*	1.85	2.07
	*R*^2^	0.99	0.95

#### Thermodynamics studies

The adsorption isotherm results were further analyzed to investigate the thermodynamics of the adsorption process. The thermodynamics equilibrium constant (*K*_*c*_) and the change in Gibbs free energy (Δ*G*°) were determined using Eqs. (14) and (15), respectively. The other parameters were estimated using a linear plot of ln *K*_*c*_ vs 1/*T*, as shown in Eq. (16). The corresponding parameters obtained from the thermodynamic studies are presented in Table 6. The negative values of Δ*G*° indicated a spontaneous adsorption process.

**Table 6:** Thermodynamics parameters for EFV and NVP adsorption onto CLDH

Pollutant	*T* (K)	Ln (*K*_c_)	Δ*G*° (kJ/mol)	Δ*H*° (kJ/mol)	Δ*S*° (J/mol·K)
Efavirenz	298	11.5	−28.49	−9.67	62.98
	308	11.35	−29.06		
	318	11.19	−29.58		
	338	11.04	−31.02		
Nevirapine	298	11.67	−28.91	−11.9	56.31
	308	11.37	−29.12		
	318	11.19	−29.58		
	338	11.08	−31.14		

Additionally, the negative values of the change in enthalpy (Δ*H*°) suggested that the adsorption process was exothermic, aligning with the findings presented in the “Interpretation of 3-dimensional (3D) and contour plots” section. Notably, the values of the change in enthalpy (Δ*H*°) were determined to be −11.14 kJ/mol for EFV and −13.91 kJ/mol for NVP, indicating that the adsorption process was predominantly governed by physisorption interactions rather than chemisorption (Alimohammady and Ghaemi 2020). It can be deduced that the adsorption of EFV and NVP onto CLDH involved relatively weaker physical interactions rather than strong chemical bonding. The positive value of Δ*S*° signified the affinity of the adsorbent for the adsorbate species. Additionally, it indicated an augmented level of randomness at the solid/solution interface, accompanied by structural modifications in both the adsorbate and the adsorbent. This phenomenon was particularly relevant to CLDH, given the structural alterations occurring during the reformation of the clay upon contact with aqueous solutions.

14 $${K}_{\textrm{c}}=\frac{1000\times {K}_{\textrm{I}}\times {MM}_{\textrm{ads}}\times {C}_{\textrm{ads}}^{{}^{\circ}}}{\gamma }$$

15 $$\Delta {G}^{{}^{\circ}}=-\textrm{R}T\ln {K}_{\textrm{c}}$$

16 $$\ln {K}_c=\frac{\Delta {S}^{{}^{\circ}}}{\textrm{R}}-\frac{\Delta {H}^{{}^{\circ}}}{\textrm{R}T}$$

where *T* is the reaction temperature (K); *R* is the universal gas constant (8.314 J/mol·K); *C*^*°*^_ads_ is the standard adsorbate concentration (1 mol/L); *K*_c_ is the thermodynamic equilibrium constant (dimensionless); *K*_I_ is the equilibrium constant depending on the best isotherm model fitted (L/mg); *MM*_ads_ is the molar mass of adsorbate (g/mol); Δ*G°* is the change in Gibbs free energy (kJ/mol); Δ*H°* is the change in enthalpy (kJ/mol); Δ*S°* is the change in entropy (kJ/mol·K); and *γ* is the activity coefficient (dimensionless);

#### Recycling of the adsorbent

**Fig. 14:**
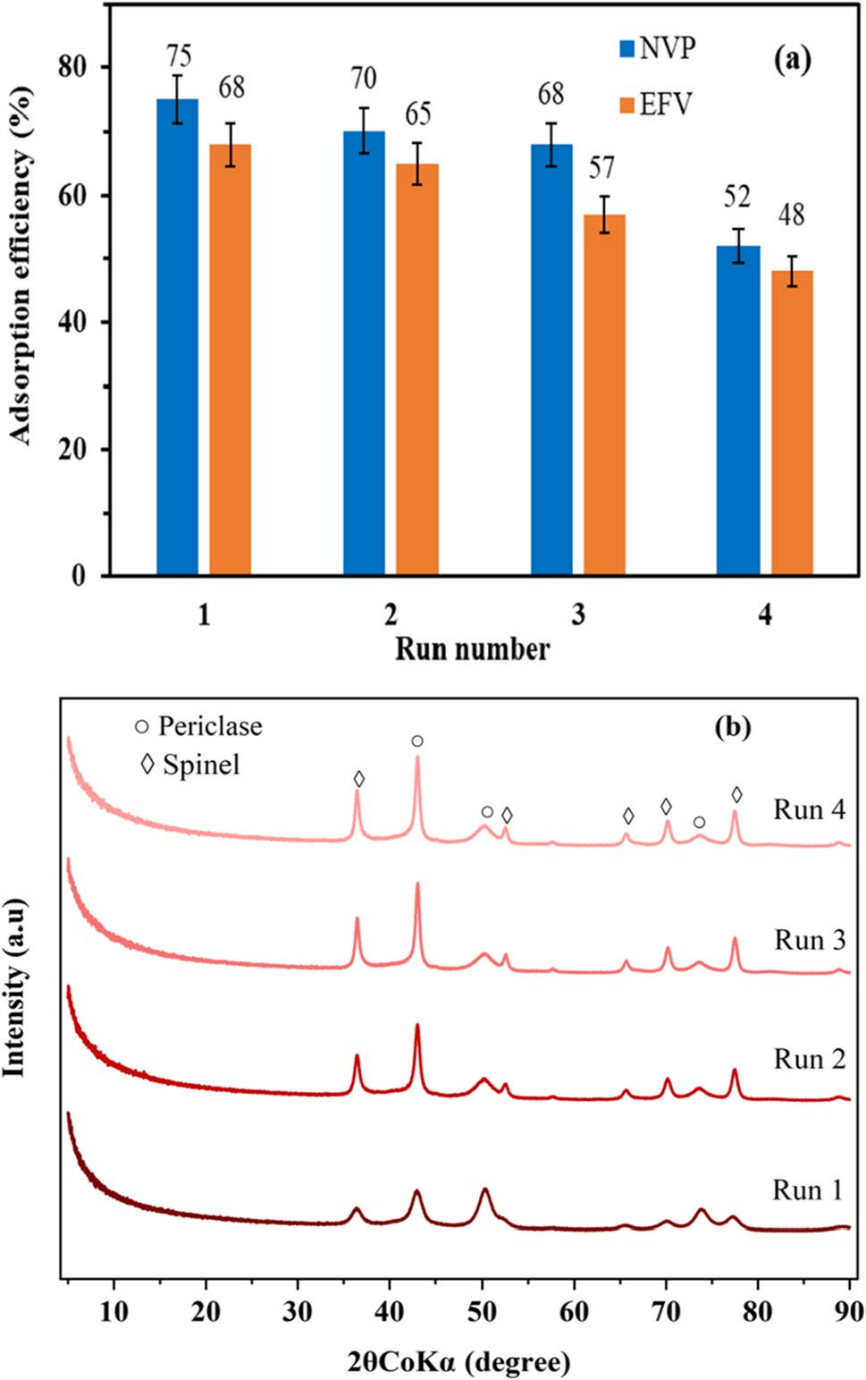
**a** Recycling and reusability of CLDH for adsorption of EFV and NVP; **b** XRD plots of recalcined LDH clay (initial concentration of 10 mg/L, pH 5, and *T* = 25 °C and an adsorbent loading of 10 g/L)

The spent clay suspensions (from both EFV and NVP adsorption) were recovered and subsequently subjected to recalcination at 500 °C for 4 h. Figure 14 a shows the adsorption efficiencies of EFV and NVP using the regenerated clay after recalcination of their respective spent CLDH. The results indicated that the decrease in EFV and NVP adsorption over the first three runs was relatively minor, amounting to approximately 11% and 7%, respectively. However, a notable decline in adsorption efficiency was observed after the fourth run, with EFV and NVP experiencing reductions of 20% and 23%, respectively. These findings suggested that the thermal regeneration of the clay for reuse was only feasible for up to three runs, after which the clay lost its sorption capacity. This observation aligned with a study by Mourid et al. (2019), who reported that LDH clay could experience a loss in its adsorption capacity upon repeated calcinations due to increased crystallinity.

Furthermore, the quantification of XRD analysis conducted on the residues from NVP adsorption experiments revealed that the proportion of the periclase phase decreased from 55% in the first run to 32% in the fourth run, as presented in Table 7. Figure 14b further supports this finding, as the diffraction peaks corresponding to the first run were characterized by broad peaks, indicative of the amorphous periclase phase. In contrast, subsequent runs exhibited sharper peaks, suggesting an increase in crystallinity and the dominance of the spinel content. It can be inferred that the same phenomenon occurred on the clay’s reusability for EFV adsorption.

**Table 7:** Phase quantification of regenerated CLDH

Run number	Periclase	Spinel
1	55.34	44.66
2	52.89	47.11
3	48.57	51.43
4	32.46	67.54

#### Computational modelling

**Fig. 15:**
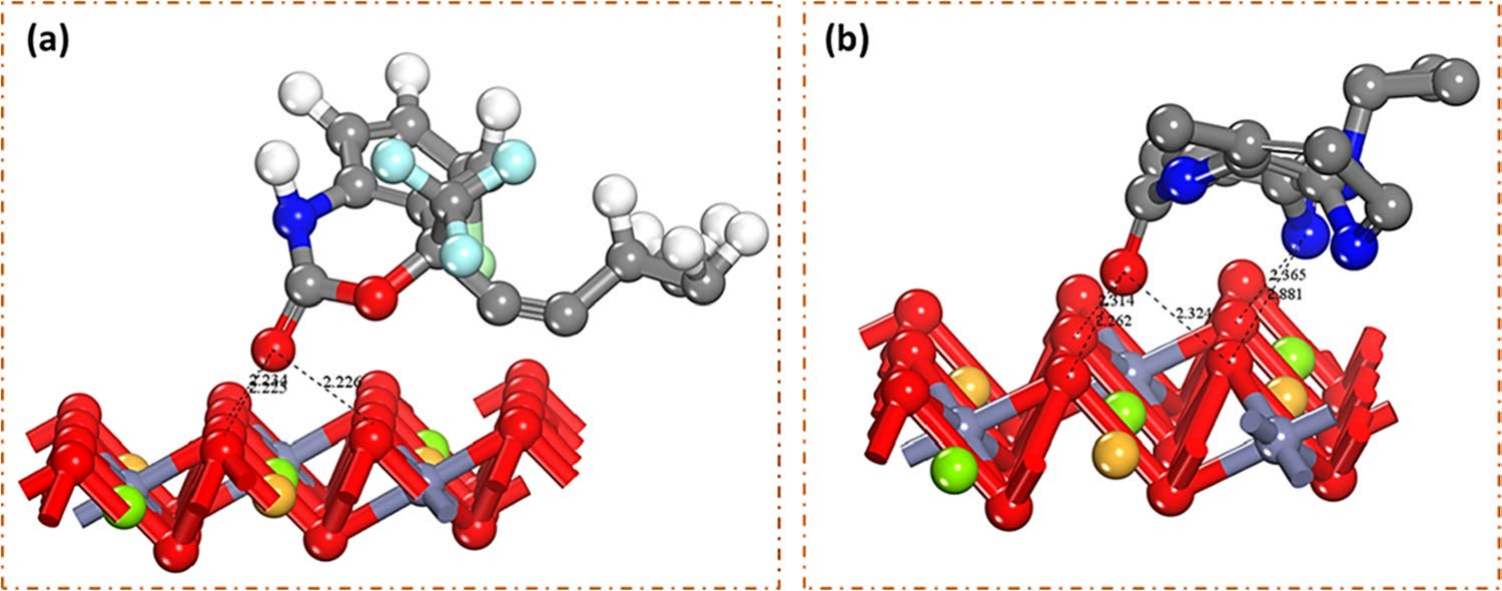
Adsorption configuration of the **a** NVP and **b** EFV on the CLDH through classical simulation

Both classical and quantum simulations were employed to examine the adsorption behavior of NVP and EFV on the CLDH. Figure 15 shows the resulting configuration of the optimized complex of NVP-CLDH and EFV-CLDH, respectively. Table 8 details the adsorption energies, total energies, and the average distances of the adsorbates from the substrate. The adsorption energy refers to the energy exchange upon adsorption of the relaxed species onto the substrate surface, a fundamental mechanism well-acknowledged in pollutant-clay interactions (Thiebault et al. 2015).

**Table 8:** Classical simulations for the adsorption of NVP and EFV on the CLDH showing the adsorption energies, total energies, and the average closest distance of the adsorbate from the substrate

Structure of complex	Adsorption energy (kcal mol^−1^)	Total energy (kcal mol^−1^)	Average distance (Å)
NVP-CLDH	−731.78	−223.17	2.30
EFV-CLDH	−512.60	−500.22	2.23

Both NVP and EFV displayed robust interactions with CLDH, as reflected in their highly negative adsorption energies. This highlighted the spontaneity of the adsorption process, which aligned with the findings from the thermodynamics studies. Furthermore, the close proximity of the adsorbates to the substrate (2.3 Å for NVP and 2.23 Å for EFV) reinforced the notion of robust adsorptive interactions. The computed adsorption energies indicated a notably stronger interaction between NVP and CLDH than EFV, with NVP exhibiting an adsorption energy of −731.78 kcal/mol compared to EFV’s −512.60 kcal/mol. This discrepancy indicated that NVP forms a more stable complex with CLDH in compared to EFV. This aligned with the reaction kinetics studies, as presented in the “Reaction kinetics and the effect of pollutant concentration” section.

**Fig. 16:**
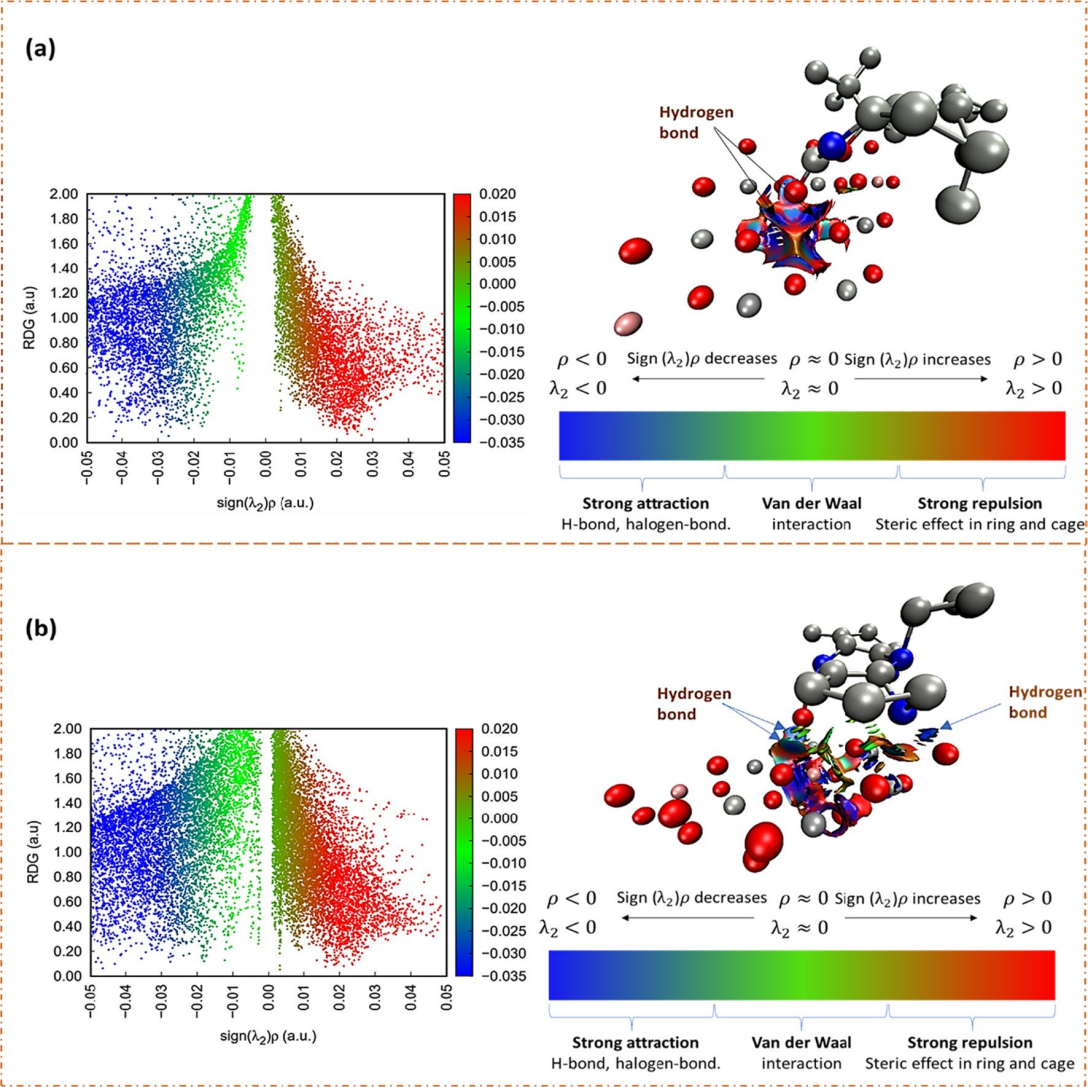
Non-covalent interaction analysis of **a** NVP and **b** EFV on the CLDH

Furthermore, the in-depth analysis involved applying the RDG and Sign(*λ*_2_)*ρ function to examine weak interaction zones. RDG isosurfaces, representing electron density and its gradient, were color-coded to denote the intensity and nature of the interactions. Blue isosurfaces indicated strong non-covalent interactions, while green represented Van der Waals interactions, and red signified strong repulsive interactions, such as steric hindrance. Identifying regions with low electron density provided valuable insights (Adekoya et al. 2022). Hence, the NCI plots (Fig. 16) visually represent the interactions between NVP, EFV, and CLDH. These plots highlighted the presence of hydrogen bonding, reiterating its role in the adsorption process. The analysis suggested he presence of dipole-dipole interactions in both NVP and EFV adsorption processes. Specifically, in NVP, the closest oxygen atom to the surface of the clay, carrying a partial negative charge (δ-), exhibited attraction towards the electropositive hydrogen atoms of the three neighboring hydroxides in CLDH, indicating the formation of a hydrogen bond.

Additionally, there was a strong attraction between the oxygen of NVP and the Mg atom in CLDH, suggesting the potential formation of an ionic bond resulting in MgO. Similarly, in EFV, the oxygen atom with a partial negative charge (δ-) experienced attraction towards the electropositive hydrogen atoms of the neighboring oxygen atoms in CLDH, forming a hydrogen bond. Furthermore, a strong attraction was observed between the oxygen of EFV and the Mg atom in CLDH, implying the potential formation of an ionic bond leading to MgO. Additionally, a hydrogen bond was observed between the nitrogen atom of EFV and one of the neighboring hydroxides in the clay structure. Therefore, the study established that both NVP and EFV formed stable complexes with CLDH, with NVP displaying a higher affinity. These interactions were predominantly driven by hydrogen bonding, as supported by the NCI plots.

## Conclusion

This study provided insightful knowledge about the adsorption of EFV and NVP on CLDH clay. These two ARVDs have been identified as persistent pollutants in waterbodies, posing potential risks to human health and the environment. The characterization of CLDH through XRD analysis revealed that it predominantly consisted of the periclase phase, which is known to be favorable for adsorption processes. This finding supported the suitability of CLDH clay as an effective adsorbent for the removal of EFV and NVP. Additionally, the investigation of the interactions between the independent adsorption variables revealed significant interactions between the solution’s initial pH and adsorbent loading, along with the interaction between the reaction temperature and the initial concentration of the pollutants. Subsequent investigations into the adsorption kinetics, isotherms, and thermodynamics revealed that the physical interactions primarily governed the adsorption process .

Additionally, the feasibility of regenerating the clay for reuse through thermal recalcination was highlighted. However, the recycling capability was limited to three runs, as the adsorption efficiency decreased significantly after that point. This decline resulted from a rise in crystallinity and a shift in phase proportions. The computational analysis reiterated that physisorption was the predominant mechanism driving the adsorption of ARVDs onto CLDH, primarily facilitated by hydrogen bonding. Furthermore, it was revealed that NVP demonstrated a higher affinity for the clay than EFV.

### Supplementary information

ESM 1 (DOCX 111 kb)

## References

[CR1] Abafe OA, Späth J, Fick J, Jansson S, Buckley C, Stark A, Pietruschka B, Martincigh BS (2018). Lc-Ms/Ms determination of antiretroviral drugs in influents and effluents from wastewater treatment plants in Kwazulu-Natal, South Africa. Chemosphere.

[CR2] Adekoya GJ, Sadiku ER, Hamam Y, Mwakikunga BW, Ray SS (2023). DFT and MC investigation of EDOT on honeycomb borophene as potential energy storage material. AIP Conference Proceedings.

[CR3] Adekoya OC, Adekoya GJ, Sadiku RE, Hamam Y, Ray SS (2022). Density functional theory interaction study of a polyethylene glycol-based nanocomposite with cephalexin drug for the elimination of wound infection. ACS Omega.

[CR4] Adeola AO, de Lange J, Forbes PB (2021). Adsorption of antiretroviral drugs, efavirenz and nevirapine from aqueous solution by graphene wool: kinetic, equilibrium, thermodynamic and computational studies. Appl Surf Sci Adv.

[CR5] Akenga P, Gachanja A, Fitzsimons MF, Tappin A, Comber S (2021). Uptake, Accumulation and impact of antiretroviral and antiviral pharmaceutical compounds in lettuce. Sci Total Environ.

[CR6] Alimohammady M, Ghaemi M (2020). Adsorptive removal of Hg^2+^ from aqueous solutions using amino phenyl-pyrazole-functionalized graphene oxide. Carbon Lett.

[CR7] Alimohammady M, Jahangiri M, Kiani F, Tahermansouri H (2017). Highly efficient simultaneous adsorption of Cd(II), Hg(II) and As(III) ions from aqueous solutions by modification of graphene oxide with 3-aminopyrazole: central composite design optimization. New J Chem.

[CR8] Amamra S, Djellouli B, Elkolli H, Benguerba Y, Erto A, Balsamo M, Ernst B, Benachour D (2021). Synthesis and characterization of layered double hydroxides aimed at encapsulation of sodium diclofenac: theoretical and experimental study. J Mol Liq.

[CR9] Caldas SS, Escarrone ALV, Primel EG (2017). Pharmaceuticals and personal care products. Chromatographic Analysis of the Environment.

[CR10] Capra L, Manolache M, Ion I, Stoica R, Stinga G, Doncea SM, Alexandrescu E, Somoghi R, Calin MR, Radulescu I (2018). Adsorption of Sb (III) on oxidized exfoliated graphite nanoplatelets. Nanomaterials.

[CR11] Chabi N, Baghdadi M, Sani AH, Golzary A, Hosseinzadeh M (2020). Removal of tetracycline with aluminum boride carbide and boehmite particles decorated biochar derived from algae. Bioresour Technol.

[CR12] Clark I, Smith J, Gomes RL, Lester E (2019). Continuous synthesis of Zn_2_Al-CO_3_ layered double hydroxides for the adsorption of reactive dyes from water. J Environ Chem Eng.

[CR13] dos Santos RMM, Gonçalves RGL, Constantino VRL, da Costa LM, da Silva LHM, Tronto J, Pinto FG (2013). Removal of Acid Green 68:1 from aqueous solutions by calcined and uncalcined layered double hydroxides. Appl Clay Sci.

[CR14] dos Santos RMM, Gonçalves RGL, Constantino VRL, Santilli CV, Borges PD, Tronto J, Pinto FG (2017). Adsorption of Acid Yellow 42 dye on calcined layered double hydroxide: effect of time, concentration, pH and temperature. Appl Clay Sci.

[CR15] Eiby S, Tobler D, Nedel S, Bischoff A, Christiansen B, Hansen A, Kjaergaard H, Stipp S (2016). Competition between chloride and sulphate during the reformation of calcined hydrotalcite. Appl Clay Sci.

[CR16] Elhachemi M, Chemat-Djenni Z, Chebli D, Bouguettoucha A, Amrane A (2022). Synthesis and physicochemical characterization of new calcined layered double hydroxide Mg Zn Co Al-CO_3_; classical modeling and statistical physics of nitrate adsorption. Inorg Chem Commun.

[CR17] Fernández LP, Brasca R, Repetti MR, Attademo AM, Peltzer PM, Lajmanovich RC, Culzoni MJ (2022). Bioaccumulation of abacavir and efavirenz in rhinella arenarum tadpoles after exposure to environmentally relevant concentrations. Chemosphere.

[CR18] Gao Y, Zhang Z, Wu J, Yi X, Zheng A, Umar A, O’Hare D, Wang Q (2013). Comprehensive investigation of CO2 adsorption on Mg–Al–CO_3_ LDH-derived mixed metal oxides. J Mater Chem A.

[CR19] Gevers BR, Naseem S, Leuteritz A, Labuschagné FJ (2019). Comparison of nano-structured transition metal modified tri-metal Mg-M-Al–LDHs (M= Fe, Zn, Cu, Ni, Co) prepared using co-precipitation. RSC Adv.

[CR20] Guo X, Wang J (2019). A general kinetic model for adsorption: theoretical analysis and modeling. J Mol Liq.

[CR21] Insani WN, Qonita NA, Jannah SS, Nuraliyah NM, Supadmi W, Gatera VA, Alfian SD, Abdulah R (2020). Improper disposal practice of unused and expired pharmaceutical products in indonesian households. Heliyon.

[CR22] Jie Z, Yichen J, Ping L, Yang L, Huiyuan T, Xiuhong D, Zehua W, Xianying D, Chunguang L, Jiehu C (2022) Rational construction and understanding the effect of metal cation substitution of three novel ternary Zn-Co–Ni-LDHs from 2d to 3d and its enhanced adsorption properties for Mo. Environ Sci Pollut Res:1–19 10.1007/s11356-022-22303-6 35945322

[CR23] Kazeem TS, Zubair M, Daud M, Mu’azu ND, Al-Harthi MA (2019). Graphene/ternary layered double hydroxide composites: efficient removal of anionic dye from aqueous phase. Korean J Chem Eng.

[CR24] Kowlaser S, Barnhoorn I, Wagenaar I (2022). Developmental abnormalities and growth patterns in juvenile Oreochromis mossambicus chronically exposed to efavirenz. Emerg Contam.

[CR25] Lei X, Jin M, Williams GR (2014). Layered double hydroxides in the remediation and prevention of water pollution. Energy Environ Focus.

[CR26] Li B, Zhang Y, Zhou X, Liu Z, Liu Q, Li X (2016). Different dye removal mechanisms between monodispersed and uniform hexagonal thin plate-like MgAl–CO_3_^2-^-LDH and its calcined product in efficient removal of Congo Red from water. J Alloys Compd.

[CR27] Li SS, Jiang M, Jiang TJ, Liu JH, Guo Z, Huang XJ (2017). Competitive adsorption behavior toward metal ions on nano-Fe/Mg/Ni ternary layered double hydroxide proved by XPS: evidence of selective and sensitive detection of Pb(II). J Hazard Mater.

[CR28] Madikizela LM, Tavengwa NT, Chimuka L (2017). Status of pharmaceuticals in African water bodies: occurrence, removal and analytical methods. J Environ Manag.

[CR29] Mascolo G, Balest L, Cassano D, Laera G, Lopez A, Pollice A, Salerno C (2010). Biodegradability of pharmaceutical industrial wastewater and formation of recalcitrant organic compounds during aerobic biological treatment. Bioresour Technol.

[CR30] Mourid EH, Lakraimi M, Benaziz L, Elkhattabi EH, Legrouri A (2019). Wastewater treatment test by removal of the sulfamethoxazole antibiotic by a calcined layered double hydroxide. Appl Clay Sci.

[CR31] Naseem S, Gevers B, Boldt R, Labuschagné FJW, Leuteritz A (2019). Comparison of transition metal (Fe, Co, Ni, Cu, and Zn) containing tri-metal layered double hydroxides (LDHs) prepared by urea hydrolysis. RSC Adv.

[CR32] Ncube S, Madikizela LM, Chimuka L, Nindi MM (2018). Environmental fate and ecotoxicological effects of antiretrovirals: a current global status and future perspectives. Water Res.

[CR33] Oliveira EL, Grande CA, Rodrigues AE (2008). CO_2_ sorption on hydrotalcite and alkali-modified (K and Cs) hydrotalcites at high temperatures. Sep Purif Technol.

[CR34] Parolini M, Pedriali A, Binelli A (2013). Application of a biomarker response index for ranking the toxicity of five pharmaceutical and personal care products (PPCPs) to the bivalve Dreissena polymorpha. Arch Environ Contam Toxicol.

[CR35] Paut KM, Tomas A, Sabo A (2016). Disposal of unused drugs: knowledge and behavior among people around the world. Rev Environ Contam Toxicol.

[CR36] Prasse C, Schlüsener MP, Schulz R, Ternes TA (2010). Antiviral drugs in wastewater and surface waters: a new pharmaceutical class of environmental relevance?. Environ Sci Technol.

[CR37] Ruan X, Huang S, Chen H, Qian G (2013). Sorption of aqueous organic contaminants onto dodecyl sulfate intercalated magnesium iron layered double hydroxide. Appl Clay Sci.

[CR38] Schoeman C, Mashiane M, Dlamini M, Okonkwo O (2015). Quantification of selected antiretroviral drugs in a wastewater treatment works in South Africa using GC-TOFMS. J Chromatogr Sep Tech.

[CR39] Soustelle M (2011). An introduction to chemical kinetics.

[CR40] Tabana L, Tichapondwa S, Labuschagne F, Chirwa E (2020). Adsorption of phenol from wastewater using calcined magnesium-zinc-aluminium layered double hydroxide clay. Sustainability.

[CR41] Tambosi JL, Yamanaka LY, José HJ, Moreira RDFPM, Schröder HF (2010). Recent research data on the removal of pharmaceuticals from sewage treatment plants (STP). Química Nova.

[CR42] Thiebault T, Guégan R, Boussafir M (2015). Adsorption mechanisms of emerging micro-pollutants with a clay mineral: case of tramadol and doxepine pharmaceutical products. J Colloid Interface Sci.

[CR43] Wooding M, Rohwer ER, Naudé Y (2017). Determination of endocrine disrupting chemicals and antiretroviral compounds in surface water: a disposable sorptive sampler with comprehensive gas chromatography–time-of-flight mass spectrometry and large volume injection with ultra-high performance liquid chromatography–tandem mass spectrometry. J Chromatogr A.

[CR44] Yang Z, Wang F, Zhang C, Zeng G, Tan X, Yu Z, Zhong Y, Wang H, Cui F (2016). Utilization of LDH-based materials as potential adsorbents and photocatalysts for the decontamination of dyes wastewater: a review. RSC Adv.

[CR45] Yin N, Wang K, Wang L, Li Z (2016). Amino-functionalized MOFs combining ceramic membrane ultrafiltration for Pb (II) removal. Chem Eng J.

[CR46] Zhang J (2019). Physical insights into kinetic models of adsorption. Sep Purif Technol.

[CR47] Zitha AB, Ncube S, Mketo N, Nyoni H, Madikizela LM (2022) Antiretroviral drugs in water: an African challenge with Kenya and South Africa as hotspots and plausible remediation strategies. Chem Afr 1-17. 10.1007/s42250-022-00417-1

