# Supplementary material for: Integrated study of antiretroviral drug adsorption onto calcined layered double hydroxide clay: experimental and computational analysis
Source: Environ Sci Pollut Res Int. 2024 Apr 22;31(22):32282–300. doi: 10.1007/s11356-024-33406-7 (PMC11133027; doi:10.1007/s11356-024-33406-7)
Supplement: Supplementary file 1 — (DOCX 111 kb) [file 11356_2024_33406_MOESM1_ESM.docx]

Integrated Study of Antiretroviral Drugs Adsorption onto Calcined Layered Double Hydroxide Clay: Experimental and Computational Analysis

Lehlogonolo S. Tabana*^, a^, Gbolahan J. Adekoya^b^ and Shepherd M. Tichapondwa^a^

^a^Department of Chemical Engineering, Sustainable Environmental and Water Utilisation Processes Division, University of Pretoria, Pretoria, South Africa.

^b^Institute of NanoEnginieering Research (INER) & Department of Chemical, Metallurgical and Materials Engineering, Faculty of Engineering and the Built Environment, Tshwane University of Technology, Pretoria, South Africa

tabana.ls@tuks.co.za

**Supplementary data**


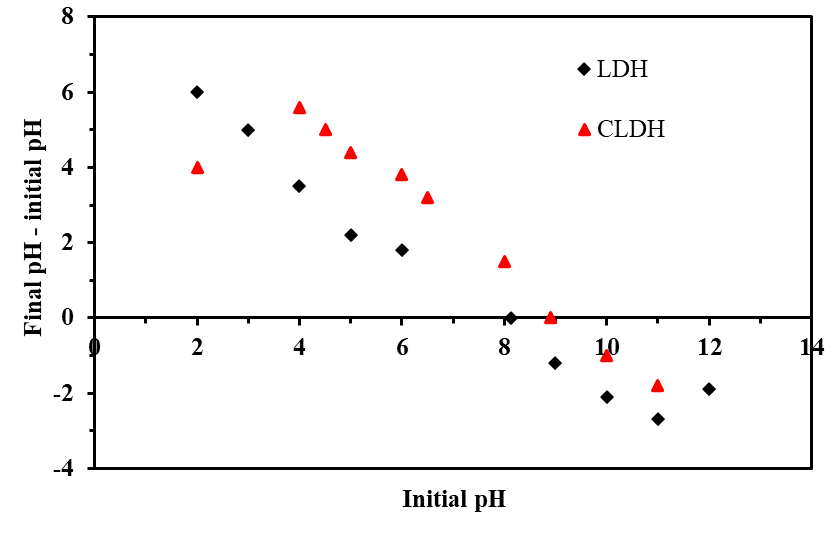


Figure S1: Point of zero charge for LDH and CLDH


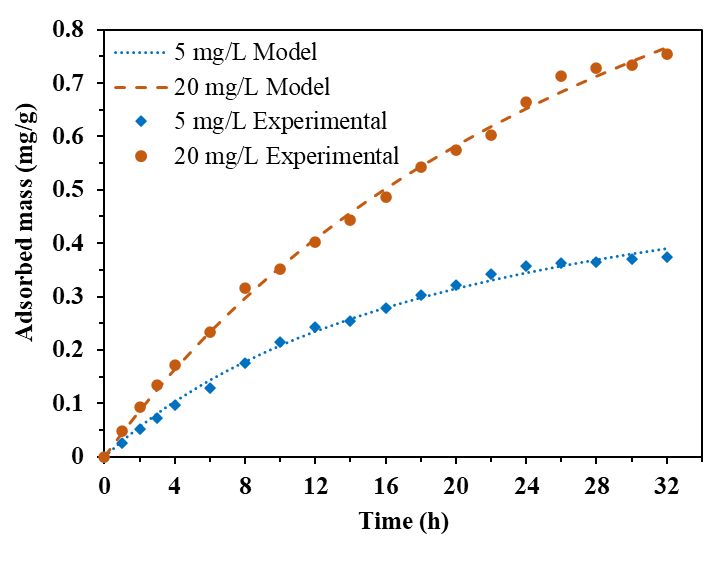


Figure S2: PSO kinetics model for EFV at initial concentrations of 5 and 20 mg/L, pH 5, T=25 ℃ and a dosage of 10 g/L

Figure S3: PSO kinetics model for NVP at initial concentrations of 5 and 20 mg/L, pH 5, T=25 ℃ and a dosage of 10 g/L

Table S 1: PFO kinetic models’ parameters

| **Concentration** | **Parameter** | **Efavirenz** | **Nevirapine** |
| --- | --- | --- | --- |
| 5 mg/L | *k_1_* (h^–1^) | 0.107 | 0.115 |
|  | *q_e_* (calculated)(mg/g) | 0.413 | 0.528 |
|  | *q_e_* (experimental)(mg/g) | 0.362 | 0.42 |
|  | *R^2^* | 0.92 | 0.90 |
|  |  |  |  |
| 10 mg/L | *k_1_* (h^–1^) | 0.104 | 0.126 |
|  | *q_e_* (calculated)(mg/g) | 0.719 | 1 |
|  | *q_e_* (experimental)(mg/g) | 0.629 | 0.748 |
|  | *R^2^* | 0.93 | 0.95 |
|  |  |  |  |
| 20 mg/L | *k_1_* (h^–1^) | 0.137 | 0.105 |
|  | *q_e_* (calculated)(mg/g) | 0.789 | 0.996 |
|  | *q_e_* (experimental)(mg/g) | 0.64 | 0.824 |
|  | *R^2^* | 0.95 | 0.93 |
